# Dietary Soluble and Insoluble Fiber With or Without Enzymes Altered the Intestinal Microbiota in Weaned Pigs Challenged With Enterotoxigenic *E. coli* F18

**DOI:** 10.3389/fmicb.2020.01110

**Published:** 2020-05-27

**Authors:** Qingyun Li, Xiyu Peng, Eric R. Burrough, Orhan Sahin, Stacie A. Gould, Nicholas K. Gabler, Crystal L. Loving, Karin S. Dorman, John F. Patience

**Affiliations:** ^1^Department of Animal Science, Iowa State University, Ames, IA, United States; ^2^Department of Statistics, Iowa State University, Ames, IA, United States; ^3^Department of Veterinary Diagnostic and Production Animal Medicine, Iowa State University, Ames, IA, United States; ^4^Agricultural Research Service of the United States Department of Agriculture-National Animal Disease Center, Ames, IA, United States; ^5^Department of Genetics, Development, and Cell Biology, Iowa State University, Ames, IA, United States

**Keywords:** *E. coli* infection, gut microbiota, insoluble fiber, soluble fiber, carbohydrases, swine, volatile fatty acids

## Abstract

Post-weaning diarrhea caused by enterotoxigenic *E. coli* (ETEC) causes significant economic losses for pig producers. This study was to test the hypotheses that an ETEC challenge disrupts intestinal microbial homeostasis and the inclusion of dietary soluble (10% sugar beet pulp) or insoluble fiber (15% corn distillers dried grains with solubles) with or without exogenous carbohydrases will protect or restore the gut microbial homeostasis in weaned pigs. Sixty crossbred piglets (6.9 ± 0.1 kg) were blocked by body weight and randomly assigned to one of six treatments (*n* = 10), including a non-challenged control (NC), ETEC F18-challenged positive control (PC), ETEC-challenged soluble fiber without (SF-) or with carbohydrases (SF+), and ETEC-challenged insoluble fiber without (IF-) or with carbohydrases (IF+). Pigs were housed individually and orally received either ETEC inoculum or PBS-sham inoculum on day 7 post-weaning. Intestinal contents were collected on day 14 or 15. The V4 region of the bacterial 16S rRNA was amplified and sequenced. High-quality reads (total 6,671,739) were selected and clustered into 3,330 OTUs. No differences were observed in α-diversity among treatments. The ileal microbiota in NC and PC had modest separation in the weighted PCoA plot; the microbial structures were slightly altered by SF+ and IF- compared with PC. The PC increased ileal *Escherichia-Shigella* (*P* < 0.01) and numerically decreased *Lactobacillus* compared to NC. Predicted functional pathways enriched in the ileal microbiota of PC pigs indicated enhanced activity of Gram-negative bacteria, in agreement with increased *Escherichia-Shigella*. The SF+ tended to decrease (*P* < 0.10) ileal *Escherichia-Shigella* compared to PC. Greater abundance of ileal *Streptococcus*, *Turicibacter*, and *Roseburia* and colonic *Prevotella* were observed in SF- and SF+ than PC (*P* < 0.05). Pigs fed IF + had greater *Lactobacillus* and *Roseburia* than PC pigs (*P* < 0.05). The ETEC challenge reduced total volatile fatty acid (VFA) compared with NC (*P* < 0.05). The SF+ tended to increase (*P* < 0.10) and SF- significantly increased (*P* < 0.05) colonic total VFA compared with PC. Collectively, ETEC challenge disrupted gut microbial homeostasis and impaired microbial fermentation capacity. Soluble fiber improved VFA production. Dietary fiber and carbohydrases altered microbiota composition to maintain or restore microbial homeostasis.

## Introduction

Enterotoxigenic *E. coli* (ETEC) is the main pathogenic bacterium inducing post-weaning diarrhea (PWD) in pigs, causing economic losses due to increased mortality, morbidity, medication cost, and decreased growth performance (Fairbrother and Gyles, 2012). Virulence factors of ETEC include the expression of fimbria (e.g., F4 or F18) and the production of enterotoxins (e.g., heat labile and/or heat stable toxins) (Zhang et al., 2007). Following adherence of fimbria through specific receptors and colonization on intestinal epithelia, the synthesized enterotoxins can be translocated into enterocytes. This leads to cellular response and subsequent increases in secretion and reductions in absorption of electrolytes and water, resulting in diarrhea (Jensen et al., 2012). In pigs, F4 and F18 are the predominant fimbrial types of ETEC strains associated with PWD (Frydendahl, 2002; Zhang et al., 2007). The expression of fimbrial adhesin binding receptors in the small intestine of pigs determines their genetic susceptibility to ETEC F18-induced diarrhea (Frydendahl et al., 2003).

With the use of antimicrobials in animal production being restricted, alternative nutritional strategies are needed to control gastrointestinal bacterial infection such as PWD and improve piglet health. Feeding dietary fiber to young pigs has gained more interest in recent years due to its functional properties, such as improvement in intestinal microbial balance (Molist et al., 2014). However, inconsistent results have been reported regarding the impact of dietary fiber on PWD in weaned pigs; this may be due to differences in the characteristics and inclusion levels of fiber, the composition of basal diets, the severity of the ETEC challenge, and the genetics and health status of pigs (Hopwood et al., 2004; Montagne et al., 2004; Wellock et al., 2008; Molist et al., 2010). Thus, the impact of the inclusion of different sources of dietary fiber in ETEC-challenged pigs fed a corn-soybean meal based diet and the associated modes of action need to be further explored.

Sugar beet pulp (SBP) and corn distillers dried grains with solubles (DDGS) are industrial coproducts that are widely available and have been used in weaned pig diets (Thomson et al., 2012; Yan et al., 2017). Sugar beet pulp is a soluble and highly fermentable fiber, with the soluble fiber mainly from pectin (uronic acid and arabinose; Serena and Knudsen, 2007). Sugar beet pulp has been shown to increase *Lactobacillus* count and improve the health status of piglets at weaning (Schiavon et al., 2004; Thomson et al., 2012; Yan et al., 2017). The fiber in corn DDGS is primarily insoluble and poorly fermentable, with arabinoxylan and cellulose being the major non-starch polysaccharides. Exogenous carbohydrases can degrade dietary fiber to release oligosaccharides or monosaccharides in the small intestine (Laerke et al., 2015; Pedersen et al., 2015). The liberated oligosaccharides in turn may stimulate the growth of beneficial bacteria and inhibit the proliferation and colonization of pathogenic bacteria (Broekaert et al., 2011; Strube et al., 2015).

Pathogenic bacterial challenges cause overgrowth of the infected bacteria and disrupt intestinal microbial homeostatis (Burrough et al., 2017; Argüello et al., 2018). The emergence of 16S rRNA high throughput sequencing technology provides an opportunity to study complex microbial communities with high resolution. Currently, limited research has investigated the effect of an ETEC challenge on intestinal microbiota of weaning pigs (Pollock et al., 2018; Massacci et al., 2020). To our knowledge, there is also no information available on the impact of feeding SBP and DDGS on gut microbiota in weaned pigs challenged with an ETEC F18.

Previous results have shown that an ETEC challenge increased the incidence of diarrhea, induced intestinal inflammation, and reduced tight junction protein gene transcription, resulting in depressed growth performance in weaned pigs (Li et al., 2019). In the same study, pigs fed diets supplemented with SBP and carbohydrases had improved growth compared to those fed the ETEC-challenged control based primarily on corn and soybean meal without fibrous coproducts or carbohydrases; this was likely due in part to a reduction in markers of inflammation. In contrast, the addition of corn DDGS without added enzymes increased the incidence of diarrhea and *E. coli* shedding compared to the ETEC-challenged control (Li et al., 2019). Clearly, more detailed information on the modulatory effects of soluble or insoluble fiber without or with carbohydrases on gut microbiota profile and function of pigs after an ETEC challenge will provide a better understanding of mechanisms by which dietary changes mitigate or exacerbate the pig’s response to the infection.

Therefore, the objectives of this study were to test the hypotheses that an ETEC F18 challenge would disrupt intestinal microbial homeostasis and that the inclusion of dietary soluble (SF; 10% SBP) or insoluble fiber (IF; 15% DDGS) with or without exogenous carbohydrases would maintain or restore microbial homeostasis in weaned pigs.

## Materials and Methods

### Animals, Diets and Experimental Design

Sixty individually housed weaned pigs (approximately 23-day old; 30 barrows and 30 gilts; average weight = 6.9 ± 0.1 kg; L337 × Camborough; PIC Inc., Hendersonville, TN) were blocked by initial body weight and randomly assigned to 1 of 6 treatments (*n* = 10 per treatment with 5 barrows and 5 gilts): a non-challenged negative control (NC), an ETEC-challenged positive control (PC), the PC fed a diet containing soluble fiber, either without or with carbohydrases (SF- and SF+, respectively), and the PC fed a diet containing insoluble fiber without or with carbohydrases (IF- and IF +, respectively). The carbohydrases contained 0.01% xylanase (Econase XT), 0.001% β-glucanase (Econase GT P), and 0.01% pectinase (Pectinase ABE), based on the manufacturer’s recommendations (AB Vista, Plantation, FL). The analyzed enzyme activities were 190,000 BXU/g xylanase, 2,320,000 BU/g β-glucanase, and 560 PE/g pectinase.

The control diet fed to NC and PC was based primarily on corn and soybean meal with 13.5% milk whey powder. The SF (10% sugar beet pulp; SBP) and IF (15% corn distillers dried grains with solubles; DDGS) were added to the control diet in place of cornstarch. Pelleted SBP was ground to similar particle size as DDGS using a 2.5 mm screen to avoid the confounding effect of different particle size among fiber sources. All diets were formulated to meet or exceed NRC (2012) estimates of requirements of weaned pigs and did not contain antibiotics or pharmacological levels of copper or zinc. Ingredient and nutrient composition of diets are presented in Supplementary Tables S1, S2. Pigs were fed *ad libitum* and had free access to water throughout the 14-day experiment.

On day 7 post weaning, pigs were orally gavaged with either freshly grown ETEC F18 inoculum (approximately 3.5 × 10^9^ cfu/mL; 6 mL per pig) or a sham inoculum of phosphate-buffered saline (PBS; 6 mL). A hemolytic ETEC F18 strain expressing heat-labile (LT), heat-stable b (STb), and enteroaggregative *Escherichia coli* heat-stable enterotoxin 1 (EAST1) was previously recovered from the intestine of a nursery pig with enteric colibacillosis and was used in this study to prepare the bacterial inoculum at the Veterinary Diagnostic Lab of Iowa State University (Ames, IA). The sows and piglets used in this experiment were not vaccinated against *E. coli* before this trial. None of the pigs shed hemolytic *E. coli* on day 0 upon arrival. All ETEC challenged pigs were confirmed to be genetically susceptible to ETEC F18 by genotype sequencing according to Frydendahl et al. (2003).

### Sample Collection

On day 7 post weaning and before inoculation, fecal samples were collected either directly from the rectum or at defecation in pigs that voluntarily eliminated feces after collecting fecal swabs for evaluation of hemolytic *E. coli* shedding prior to challenge. Fecal samples were immediately snap-frozen in liquid N and stored at -80°C pending DNA extraction. On each of days 14 and 15, half of the pigs (5 pigs per treatment) were euthanized by captive bolt stunning followed by exsanguination. Post-euthanasia, the abdomen was opened and the entire gastrointestinal tract was removed. Approximately 2–5 g of digesta from the ileum (30 cm from the ileal-cecal junction) and mid-colon were collected into tubes, immediately snap-frozen in liquid N, and stored at -80°C pending DNA extraction. The pH of ileal, cecal, and colonic digesta was measured by directly inserting the probe of a portable pH meter (Oakton Instruments, Vernon Hills, IL, United States) into the contents following mixing. The digesta (3–5 g) of the cecum and colon were also collected and stored at −20°C pending volatile fatty acid (VFA) analysis.

### Volatile Fatty Acid Analysis

Concentration of VFA in intestinal digesta was determined using Gas Chromatography (3800 Varian GC, Agilent Technologies, Santa Clara, CA, United States). One gram of digesta samples were thawed at 4°C and mixed well followed by suspension in 2.5 mL of distilled water in a screw-capped tube. After being vortexed, 1 mL of the mixture was transferred into 1.5 mL centrifuge tubes and mixed with 0.2 mL of metaphosphoric acid to remove proteins from the digesta. Isocaproic acid (48.3 mM; Sigma-Aldrich, St. Louis, MO, United States) was used as an internal standard and 0.1 mL was added to each sample. The tubes were then centrifuged at 15, 000 × g at 4°C for 20 min. The supernatant (1 mL) was transferred into 1.5 mL GC vials and each sample was analyzed in duplicate for VFA. A flame ionization detector was used with an oven temperature of 60–200°C. The Nukol capillary column (15 m × 0.25 mm × 0.25 μm; Sigma-Aldrichı, Bellefonte, PA, United States) was operated with highly purified helium, as the carrier gas, at 1 mL/min.

### DNA Extraction and 16S Library Preparation

All intestinal digesta samples were sent to the Iowa State University Veterinary Diagnostic Laboratory for DNA extraction and subsequent 16S rRNA sequencing. Digesta samples (2–5 g) were resuspended in 30 mL PBS. Each sample (300 μL) was added to a KingFisher^TM^ Flex Purification System with MagMAX^TM^ Pathogen RNA/DNA Kit (Thermo Fisher Scientific, Waltham, MA, United States) for batch DNA extraction according to the manufacturer’s instructions. The V4 region of the 16S rRNA gene was amplified using the 515F/806R primer set (515F: GTGCCAGCMGCCGCGGTAA; 806R: GGACTACHVGGGTWTCTAAT) with AccuPrime^TM^ Pfx SuperMix (Thermo Fisher Scientific, Waltham, MA, United States). The PCR cycling program included one cycle of 95°C for 2 min, 30 cycles of 95°C for 20 s, 55°C for 15 s, and 72°C for 5 min, followed by one cycle of 72°C for 10 min. PCR products of random samples were selected to run on Qiagen Qiaxcel to confirm success of the PCR. Library cleanup was performed using Agencourt AMPure XP beads (Beckman Coulter, Inc., Brea, CA, United States) and then quantified using Kapa Library Quantification Kit (Kapa Biosystems, Wilmington, MA, United States) with QuantStudio 5 Real-Time PCR system (Thermo Fisher Scientific, Waltham, MA, United States) and pooled to single tube such that each library had equal final concentration.

### Illumina MiSeq Sequencing

The pooled library was sequenced on the Illumina MiSeq sequencing platform using V3 MiSeq cartridges to produce 2 × 250 bp paired end reads. Customized sequencing primers for read 1 (5′-TATGGT AATTGTGTGCCAGCMGCCGCGGTAA-3′), read 2 (5′-AGTCAGTCAGCCGGACTACHVGGGTWTCTAAT-3′) and index read (5′-ATTAGAWACCCBDGTAGTCCGGCTGA CTGACT-3′) were utilized during the sequencing procedure.

### Quality Filtering and Sequence Analysis

Raw data were demultiplexed based on dual indices to generate paired-end reads for each sample. Samples that failed to generate enough PCR products and contained very low numbers of sequencing reads were removed. High quality reads were selected and clustered into operational taxonomic units (OTUs) based on the workflow of Mothur MiSeq SOP (v1.39.5; Schloss et al., 2009). Several steps for pre-clustering data curation were conducted, including: (1) Paired-end reads were merged and sequences with ambiguous bases or longer than 275 bp were removed; (2) Reads were compressed to unique sequences and count tables were generated; (3) After aligning sequences to the V4 region of the 16S rRNA gene in the SILVA v128 database (Quast et al., 2012), sequences were trimmed at both ends and all unaligned sequences were removed; (4) Sequences were pre-clustered allowing up to 2 differences for denoising purposes; (5) Chimera sequences were identified and removed by the VSEARCH algorithm (Rognes et al., 2016); and, (6) Sequences that were not classified to bacteria in SILVA v128 using the Naive Bayes classifier (Wang method) with minimum confidence score of 0.8 were discarded. Finally, OTUs were clustered within 97% similarity using OptiClust (Westcott and Schloss, 2017), based on the distance matrix generated by default. Taxonomic assignment of OTUs was performed based on SILVA v128. QIIME1 (Caporaso et al., 2010) was used to convert the data format for different software packages in later analyses. A venn diagram was drawn to show similarities and differences of OTUs identified in fecal, ileal and colonic digesta samples.

### Bioinformatic Analyses

The linear discriminant analysis effect size (LEfSe) method (Galaxy v1.0; Segata et al., 2011) was used to identify biomarkers characterizing differences between groups under different conditions. Taxa with very low abundance (maximum relative abundance < 0.1% among all samples) were discarded. Samples from the ileum, colon, and feces were analyzed separately in LEfSe. After reclassifying OTUs based on the Greengene database v13.5 (DeSantis et al., 2006), the phylogenetic investigation of communities by reconstruction of unobserved states (PICRUSt, Galaxy v1.0.0; Langille et al., 2013) was used to generate predictive functional profiling of microbiota. Gene functions were collapsed into the 3rd level of the Kyoto Encyclopedia of Genes and Genomes (KEGG) Pathways. Predicted gene counts in each pathway were compared between groups using LEfSe.

### Statistical Analysis

Statistical analysis of the OTU table was performed mainly with the Phyloseq package (v1.24.2; McMurdie and Holmes, 2013) in R v3.5.0. Microbial composition analyses within groups were performed at phylum, family and genus levels. Alpha-diversity indices (Chao1, Shannon, and Inverse Simpson) were calculated and differences among treatments were compared using a pairwise t-test. Following the recommended procedures by Callahan et al. (2016), prevalence filtering (removing rare phyla with abundance ≤ 5 and non-prevalent OTUs present in < 5% samples) was conducted and abundance was normalized under even sampling depth (McMurdie and Holmes, 2014) before studying beta-diversity. Beta-diversity among treatments was compared using permutational multivariate analysis of variance (PERMANOVA; Anderson, 2001) implemented in the vegan package (v 2.5-2; Oksanen et al., 2019) based on both weighted and unweighted UniFrac distance matrices (Lozupone and Knight, 2005). *P*-values were calculated using 999 permutations. Principal coordinates analysis (PCoA; Borg and Groenen, 2005) was performed for visualization. Alpha- and beta-diversity among fecal, ileal and colonic samples were also compared with pairwise *t*-test and PERMANOVA.

Differential abundance analysis of taxa was conducted at the phylum, family and genus levels and statistical significance was assessed to evaluate the effect of ETEC challenge and dietary treatments. Taxa existing in < 10% samples were discarded. For taxa at the family and genus level, differences in abundance between groups of interest were detected using the DESeq2 package (v 1.20.0; Love et al., 2014). Geometric means were pre-calculated before estimating size factor with DESeq2. The raw count data were fitted to a generalized linear model (GLM) with a negative binomial family and a log link function in DESeq2. Preplanned contrasts were used to identify differentially abundant taxa between groups of interest and significance was assessed by Wald tests. Specific phyla with significantly different relative abundance between groups were detected using the Kruskal–Wallis (KW) non-parametric method.

Pearson correlation coefficients between VFA and most abundant genus (>1% in at least one treatment) in the colonic digesta were calculated and visualized using corrplot package (v0.84; Wei and Simko, 2017). Statistical significance of correlation coefficients was assessed by a R function cor.test provided in corrplot package. All *P*-values from multiple comparisons were adjusted by the Benjamini and Hochberg (BH) method (Benjamini and Hochberg, 1995) with a false discovery rate controlled at 5%.

The VFA and pH data were analyzed as a randomized complete block design using PROC GLIMMIX of SAS (9.4) with pen (pig) as the experimental unit. Treatment, sex, and their interaction were included in the model as fixed effects. Block was a random effect. Pre-planned contrasts were performed using the ESTIMATE statement to evaluate the effects of the ETEC challenge (NC vs. PC) and dietary treatment (SF-, SF+, IF-, or IF + vs. PC), and to compare the effect of fiber sources (SF vs. IF), without or with enzymes, as well as fiber by enzyme interactions. Treatment least square means were reported. Differences were considered significant if *P* ≤ 0.05 and tendencies if 0.05 < *P* ≤ 0.10.

## Results

### Intestinal Bacterial Richness and Diversity

**FIGURE 1:**
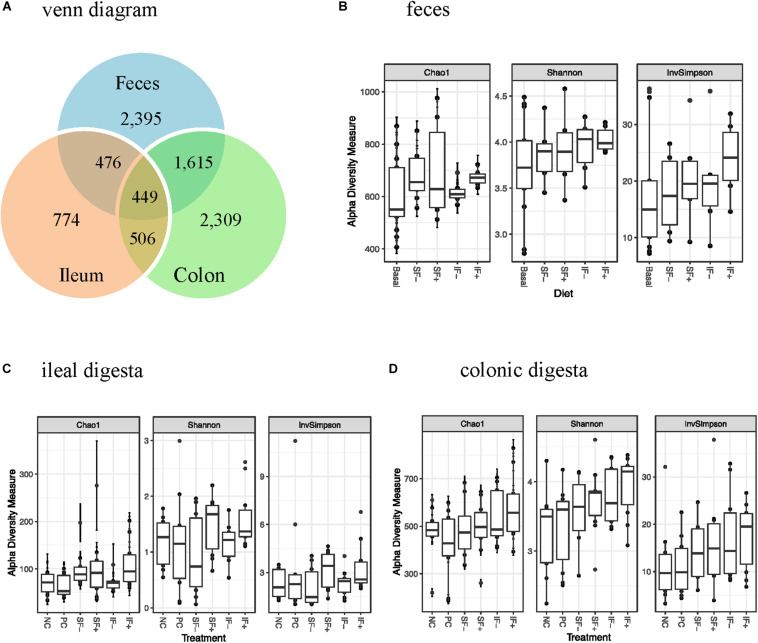
OTUs and alpha-diversity (Chao1 richness, Shannon and InvSimpson diversity indices) of bacterial community of weaned pigs challenged with ETEC. **(A)** Venn diagram showing the number of OTUs assigned in pre-challenge feces and post-challenge ileal and colonic digesta samples. **(B)** Alpha diversity in pre-challenge fecal microbiota; Basal: control diet (*n* = 13); SF-: soluble fiber without carbohydrases (*n* = 8); SF+: soluble fiber with carbohydrases (*n* = 6); IF-: insoluble fiber without carbohydrases (*n* = 6); IF+: insoluble fiber with carbohydrases (*n* = 6). **(C)** Alpha diversity in post-challenge ileal microbiota. **(D)** Alpha diversity in post-challenge colonic microbiota. NC, non-challenged control diet; PC, ETEC-challenged control diet; *n* = 10 pigs per treatment for ileal and colonic samples.

One sample from the ileum and two from the colon contained very low numbers of sequencing reads, and thus were deleted from the data analysis. There were 156 samples with 39 pre-challenge fecal, and 59 and 58 post-challenge ileal and colonic samples, respectively. A total of 6,671,739 high-quality reads were obtained after size filtering, quality control, and chimera removal, with an average of 51,080, 34,913, and 45,168 sequences per fecal, ileal and colonic sample, respectively. Based on 97% sequence similarity, 3,300 OTUs were classified, with 774 in ileal, 2,309 in colonic, and 2,395 in fecal samples (Figure 1A).

#### Alpha-Diversity

During pre-challenge period, no significant diet-induced effects were observed on Chao1 richness, Shannon or InvSimpson diversity indices in fecal microbiota (Figure 1B). Similarly, no significant differences were observed in any α-diversity indicators among treatments in ileal and colonic microbiota after the ETEC challenge (Figures 1C,D), except for a tendency for a greater Shannon diversity index in IF + than NC in the colonic microbiota (*P* < 0.10). As expected, ileal bacterial community had lower Chao1 richness, Shannon and InvSimpson indices than colonic and fecal bacteria.

#### Beta-Diversity

**FIGURE 2:**
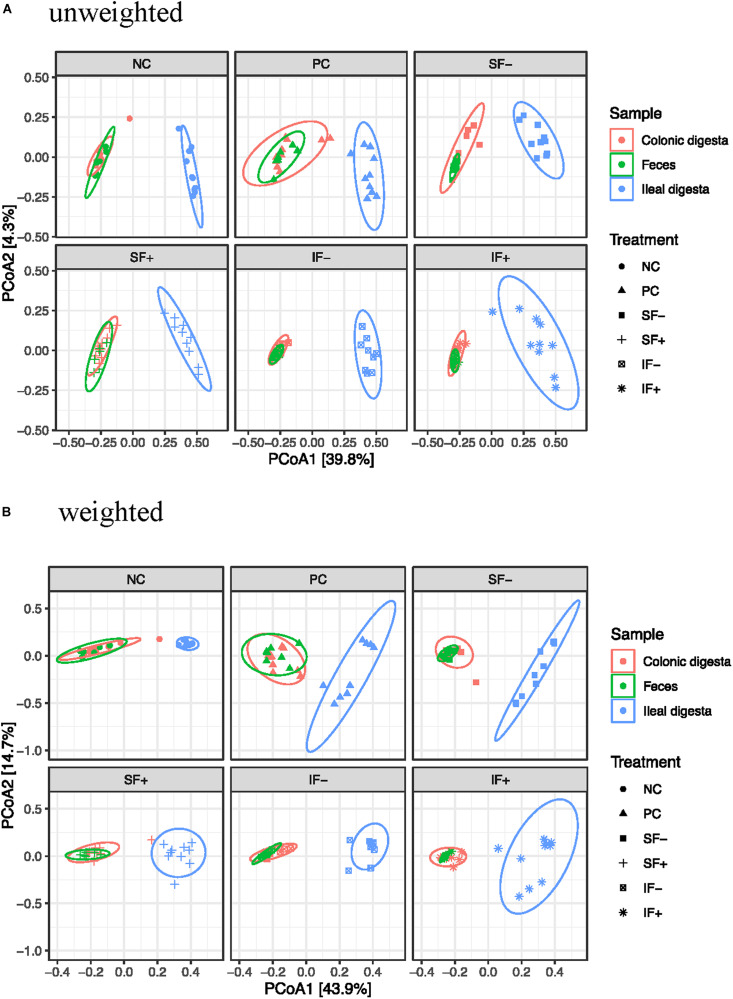
Principal coordinates analysis (PCoA) score plot of ileal, colonic, fecal samples. **(A)** Unweighted UniFrac distances. **(B)** Weighted UniFrac distances; NC, non-challenged control diet; PC, ETEC-challenged control diet; SF-, soluble fiber without carbohydrases; SF+, soluble fiber with carbohydrases; IF-, insoluble fiber without carbohydrases; IF+, insoluble fiber with carbohydrases.

Beta-diversity was assessed by PCoA based on both weighted and unweighted UniFrac distance matrices to indicate the similarity between microbial communities. The results of PERMANOVA based on weighted and unweighted UniFrac distances revealed distinct clustering patterns between the ileal and colonic microbiota (*R*^2^ = 0.40 and 0.36, respectively; *P* < 0.01; Figures 2A,B) or between ileal and fecal microbiota (*R*^2^ = 0.44 and 0.39, respectively; *P* < 0.01). Diet had no significant effect on beta-diversity of fecal microbial community during the pre-challenge period. The post-challenge ileal microbiota of pigs on NC vs. PC had modest separation with the weighted UniFrac distances (*R*^2^ = 0.31; *P* < 0.05). While the structure of the ileal microbial community was affected by SF+ and IF- (*P* < 0.05), the degree of separation was very limited according to low *R*^2^-values (0.17 and 0.22) with the weighted UniFrac distances. Similar structure of the intestinal microbiota community was found in the colon between NC vs. PC and among all 5 challenged treatments as *R*^2^-values were below 0.15, indicating overlap in community structure.

### Diets Altered Intestinal Microbiota

#### Pre-challenge Fecal Digesta

**FIGURE 3:**
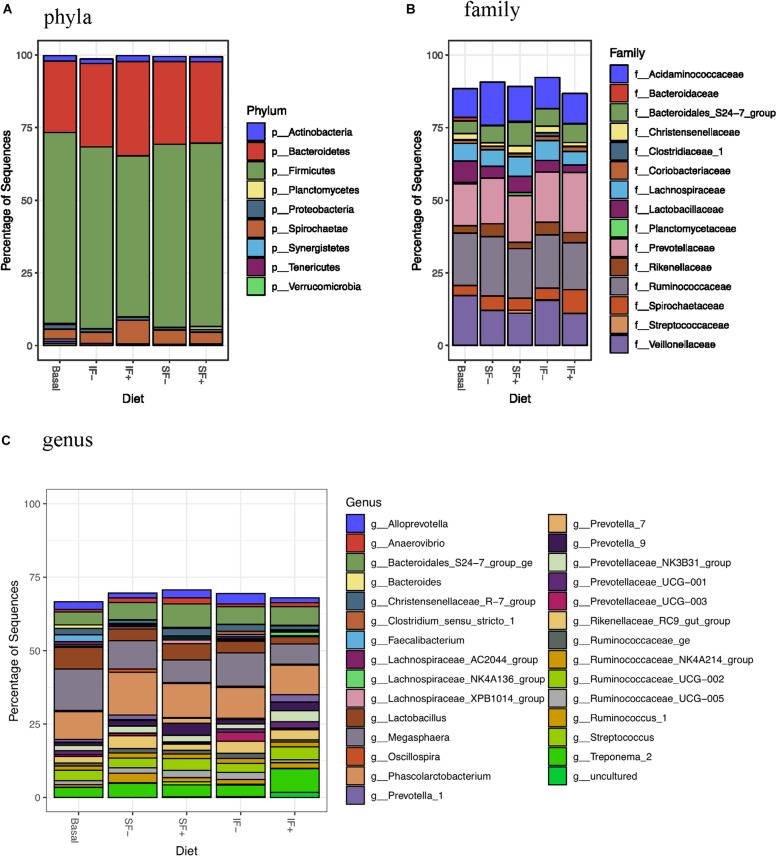
Relative abundance of bacterial taxa in feces of pigs fed control (basal; *n* = 13), soluble fiber diet without (SF-; *n* = 8) or with carbohydrases (SF+; *n* = 6), or insoluble fiber diet without (IF-; *n* = 6) or with carbohydrases (IF+; *n* = 6) before ETEC challenge. **(A)** phyla **(B)** family **(C)** genus.

At the phylum level, *Firmicutes* (>55%) and *Bacteroidetes* (>24%) dominated fecal microbiota, followed by *Saccharibacteria, Actinobacteria*, and *Proteobacteria* (Figure 3A and Supplementary Table S3). However, no significant differences in any bacterial phyla were detected among treatments. At the family level, *Ruminococcaceae*, *Prevotellaceae*, *Veillonellaceae*, and *Acidaminococcaceae* were dominant bacteria (Figure 3B and Supplementary Table S4). *Megasphaera*, *Lactobacillus*, *Ruminococcaceae UCG-002*, *Phascolarctobacterium*, and *Bacteroidales S24-7 group ge* represented dominant bacteria at the genus level (Figure 3C and Supplementary Table S5).

Individual taxa that are differentially abundant between dietary treatment and basal control diet are listed in Supplementary Table S6 (DESeq2, log2 fold change). Pigs fed IF + had decreased *Lactobacillaceae* and pigs fed SF+ had increased *Streptococcaceae* compared to those that consumed the control diet (*P* < 0.05). Additionally, lower abundance of *Bacteroidaceae* was detected in pigs fed SF- and IF- than those fed basal diets (*P* < 0.05). At the genus level, *Bacteroides* was decreased by SF- and IF- compared to the basal diet (*P* < 0.05). Additionally, pigs fed SF- had lower *Faecalibacterium* under the *Ruminococcaceae* family compared to those fed basal (*P* < 0.05). LEfSe analyses also revealed less abundant *Lactobacillaceae* in IF + pigs and more abundant *Streptococcaceae* in SF+ pigs in compariso to control pigs (Supplementary Figures S1B,D), in line with DESeq2 results.

#### Post-challenge Ileal and Colon Digesta

**FIGURE 4:**
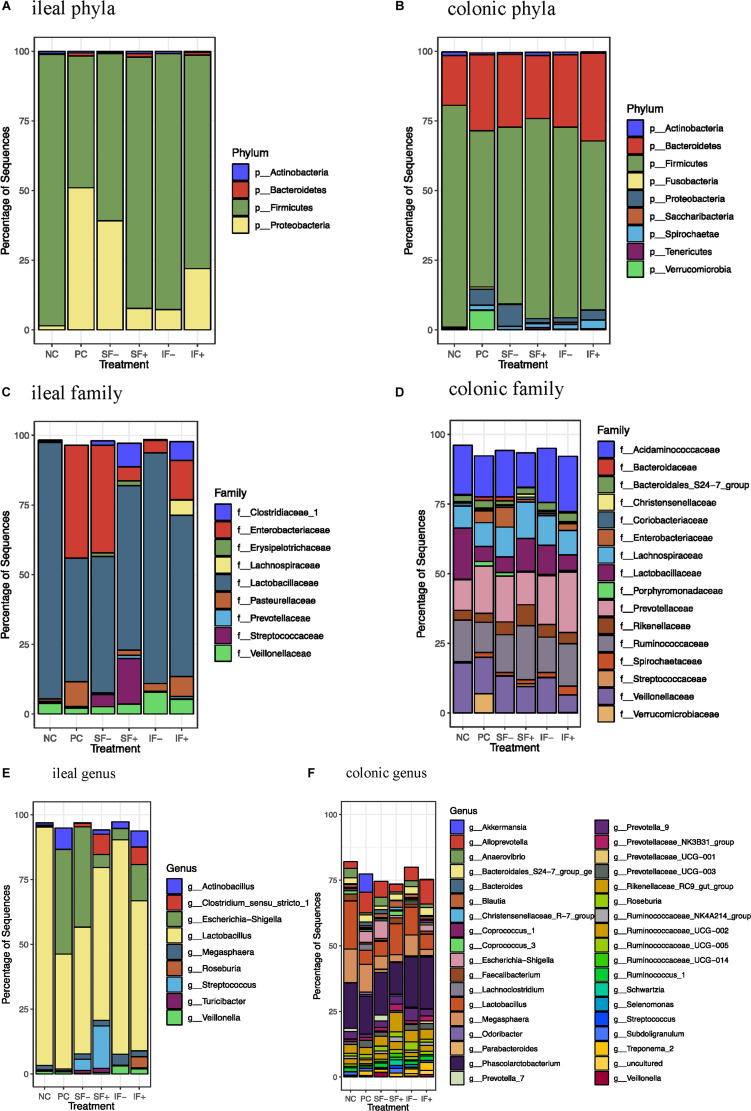
Relative abundance of bacterial taxa in ileal and colonic digesta of pigs fed control diet without ETEC (NC), control diet with ETEC (PC), soluble fiber diet without (SF-) or with carbohydrases (SF+), or insoluble fiber diet without (IF-) or with carbohydrases (IF+) after an ETEC challenge. **(A)** Ileal phyla; **(B)** Colonic phyla; **(C)** Ileal family; **(D)** Colonic family; **(E)** Ileal genus; **(F)** Colonic genus. *n* = 10 pigs per treatment except for IF- with ileal digesta from 9 pigs and SF- with colonic digesta from 8 pigs after sequence filtering.

At the phylum level, *Firmicutes* (>59%) was the most dominant in the ileal digesta of pigs fed NC, SF-, SF+, IF-, and IF + (Figure 4A and Supplementary Table S7) and in colonic digesta of all pigs, regardless of treatments (>56%; Figure 4B and Supplementary Table S8) in pigs fed NC, SF+, IF-, and IF+. For pigs fed PC, *Proteobacteria* (51.01%) was the most dominant phylum followed by *Firmicutes* (47.28%) in ileal digesta. *Bacteroidetes* was increased in the colon compared with the ileum (≥17.91% vs. 0.05%), irrespective of treatments. The ETEC challenge (PC) increased *Proteobacteria* and decreased *Firmicutes* in the ileum compared with NC (*P* < 0.05). Compared with PC, the relative abundance of *Proteobacteria* in the ileum was numerically reduced to 7.68 and 7.23% in SF+ and IF-, respectively. Similarly, SF+ and IF- numerically increased the relative abundance of ileal *Firmicutes* to a level close to that in NC. In the colon, PC increased the relative abundance of *Proteobacteria* and decreased *Firmicutes* (*P* < 0.05) and tended to decrease *Actinobacteria* (*P* < 0.10) compared with NC; SF+ and IF- numerically reduced the extent of changes in these bacterial phyla.

At the family level, *Lactobacillaceae* was the most dominant family in the ileal microbiota in all treatments and *Enterobacteriaceae* was also dominant in the ileum of pigs fed PC and SF- (Figure 4C). Colonic microbiota was dominated by *Acidaminococcaceae*, *Ruminococcaceae*, *Prevotellaceae*, *Lachnospiraceae*, *Veillonellaceae*, and *Lactobacillaceae* (Figure 4D and Supplementary Table S9). At the genus level, *Lactobacillus* dominated the ileal microbiota in NC (91.99%), and PC numerically decreased its abundance to 44.32% (Figure 4E). *Escherichia-Shigella* was the second dominant genus in the ileum of pigs fed NC and SF+. *Phascolarctobacterium*, *Lactobacillus*, and *Megasphaera* were dominant genera in the colonic digesta (Figure 4F and Supplementary Table S10).

The ETEC challenge (PC) increased ileal *Enterobacteriaceae* compared with NC (*P* < 0.01); SF+ and IF- tended to reduce (*P* < 0.10) ileal *Enterobacteriaceae* compared with PC (Supplementary Table S11). The SF+ significantly reduced ileal *Enterobacteriaceae* abundance compared with SF- (*P* < 0.05). Compared with PC, SF+ significantly increased (*P* < 0.05) and SF- and IF + tended (*P* < 0.10) to increase *Prevotellaceae* in the ileum. The addition of exogenous enzymes to the insoluble fiber diets (IF + vs. IF-) significantly increased ileal *Prevotellaceae* abundance (*P* < 0.05). Pigs fed SF, regardless of carbohydrase addition, had greater proportions of ileal *Streptococcaceae* and *Erysipelotrichaceae* than pigs in PC (*P* < 0.01). However, IF- significantly decreased ileal *Erysipelotrichaceae* compared to PC and IF + (*P* < 0.01). The addition of carbohydrases, irrespective of fiber type, increased *Clostridiaceae 1* in the ileum compared to PC and diets without carbohydrases (*P* < 0.01). Greater abundance of ileal *Lachnospiraceae* was observed in pigs fed IF + compared to those fed PC and IF- (*P* < 0.01). Pigs on SF- had lower ileal *Pasteurellaceae* compared to those on PC (*P* < 0.01). In the colon, pigs on PC had greater abundance of *Enterobacteriaceae* and lower abundance of *Lactobacillaceae* compared to those on NC (*P* < 0.05; Supplementary Table S12). The SF+ increased *Streptococcaceae* in the colon compared to PC (*P* < 0.05).

At the genus level, PC increased ileal *Escherichia-Shigella* compared with NC (*P* < 0.01; Supplementary Table S11). The SF+ tended to reduce *Escherichia-Shigella* compared to PC (*P* < 0.10) and significantly reduced *Escherichia-Shigella* compared to SF- (*P* < 0.01). Pigs fed IF + increased *Lactobacillus* compared to those fed PC (*P* < 0.05). Greater *Streptococcus*, *Clostridium sensu stricto 1*, *Turicibacter*, and *Roseburia* were observed in the ileum of pigs fed SF- and SF+ compared to those fed PC (*P* < 0.05). The SF- decreased ileal *Actinobacillus* compared with PC (*P* < 0.05). Pigs fed IF + had greater abundance of ileal *Clostridium sensu stricto* 1 and *Roseburia* than those fed PC and IF- (*P* < 0.01). In the colon, PC increased *Escherichia-Shigella* and decreased *Lactobacillus* in comparison to NC (Supplementary Table S12). In addition, PC tended to decrease *Prevotella 7* compared to NC (*P* < 0.10) and SF- increased *Prevotella 7* compared to PC (*P* < 0.05). Both SF- and SF+ decreased *Odoribacter* compared to PC (*P* < 0.01). The SF+ decreased *Veillonella* compared to PC and SF- (*P* < 0.05). The abundance of *Lachnoclostridium* was greater in pigs fed PC than all the other treatments (*P* < 0.05). A trend for lower abundance of *Prevotellaceae UCG 003* and greater abundance of *Ruminococcaceae NK4A214 group, Ruminococcaceae UC 014* and *Streptococcus* in the colon was observed in SF+ compared to PC (*P* < 0.10). The IF- tended to decrease colonic *Bacteroides* compared to PC (*P* < 0.10).

**FIGURE 5:**
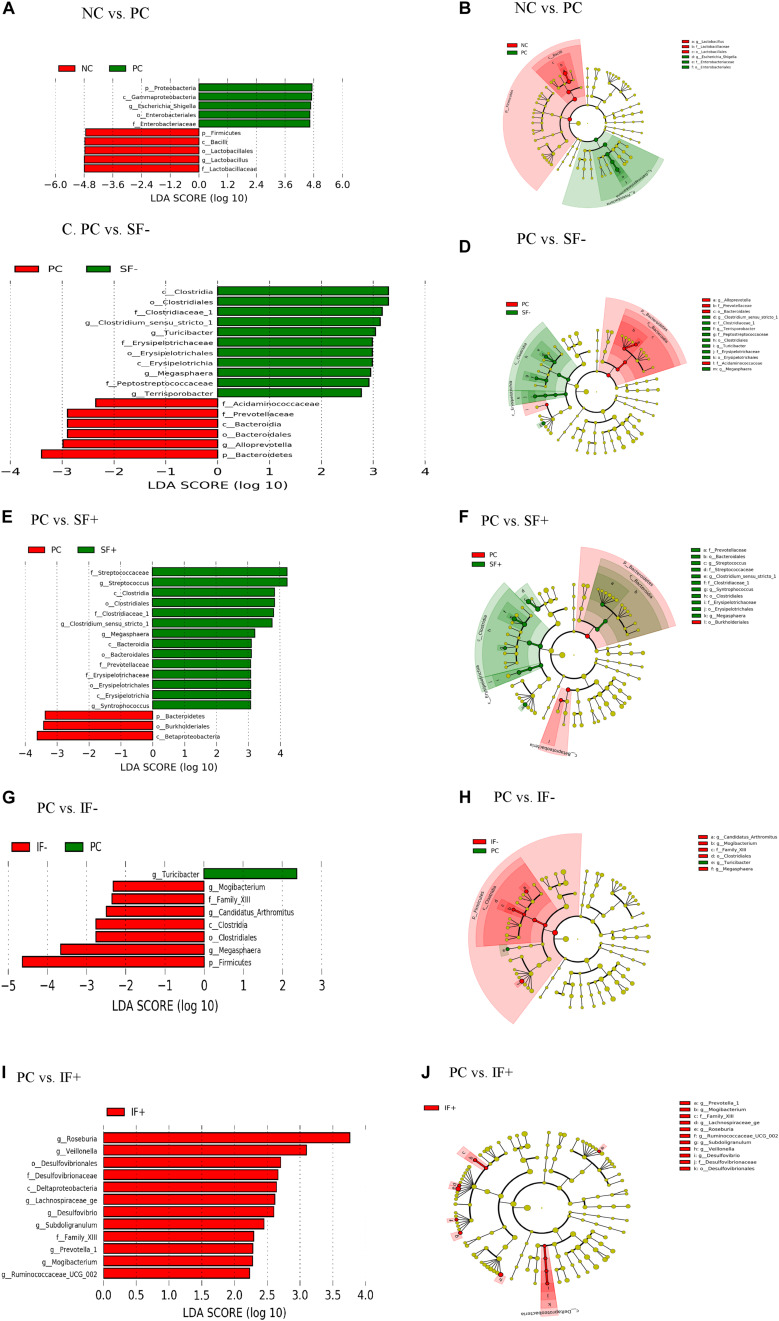
LEfSe reveals predicted biological effect sizes of differential taxa in ileal microbiota of pigs fed control diet without ETEC (NC), control diet with ETEC (PC), soluble fiber diet without (SF-) or with carbohydrases (SF+), or insoluble fiber diet without (IF-) or with carbohydrases (IF+) after an ETEC challenge. Histogram **(A,C,E,G,I)** of the linear discriminant analysis (LDA) scores computed for features differentially abundant bacteria and Cladogram **(B,D,F,H,J)** revealing statistically and biologically consistent differences in detected taxa between **(A,B)** NC and PC; **(C,D)** PC and SF-; **(E,F)** PC and SF+; **(G,H)** PC and IF-; and **(I,J)** PC and IF+.

In addition, LEfSe revealed ileal and colonic *Escherichia-Shigella* was enriched in PC pigs and *Lactobacillus* was enriched in NC pigs (Figures 5A,B and Supplementary Figures S2A,B). Enriched *Clostridium sensu stricto* 1, *Turicibacter, Megasphaera, and Terrisporobacter* in the ileum and *Prevotella 7, Rumminococcus 1, Ruminococcaceae UCG 014* in the colon were observed in pigs fed SF- compared to PC pigs (Figures 5C,D and Supplementary Figures S2C,D). *Streptococcus*, *Clostridium sensu stricto 1*, *Megasphaera*, *Erysipelotrichaceae*, and *Prevotellaceae* were differentially abundant bacterial taxa in ileal digesta of pigs fed SF+ compared to pigs fed PC (Figures 5E,F). In the colon, *Ruminococcaceae UCG 014* and *Ruminococcaceae NK4A214* group under the *Ruminococcaceae* and *Blautia* were enriched in SF+ pigs compared to PC pigs (Supplementary Figures S2E,F). Pigs fed IF- had enriched *Mogibacterium* and *Megasphaera* in the ileum and *Candidatus Saccharimonas* in the colon compared to PC pigs (Figures 5G,H and Supplementary Figures S2G,H). The SF+ pigs had more abundant *Roseburia, Veillonella*, *Desulfovibrio*, *Lachnospiraceae g*e, *Prevotella 1*, and *Ruminococcaceae UCG 002* in the ileum, and *Ruminococcaceae* family and *Prevotella 1* and *Prevotella 9* in the colon compared to PC pigs (Figures 5I,J and Supplementary Figures S2I,J).

### Predicted Functional Capacity of the Ileal Digesta Microbiota

**FIGURE 6:**
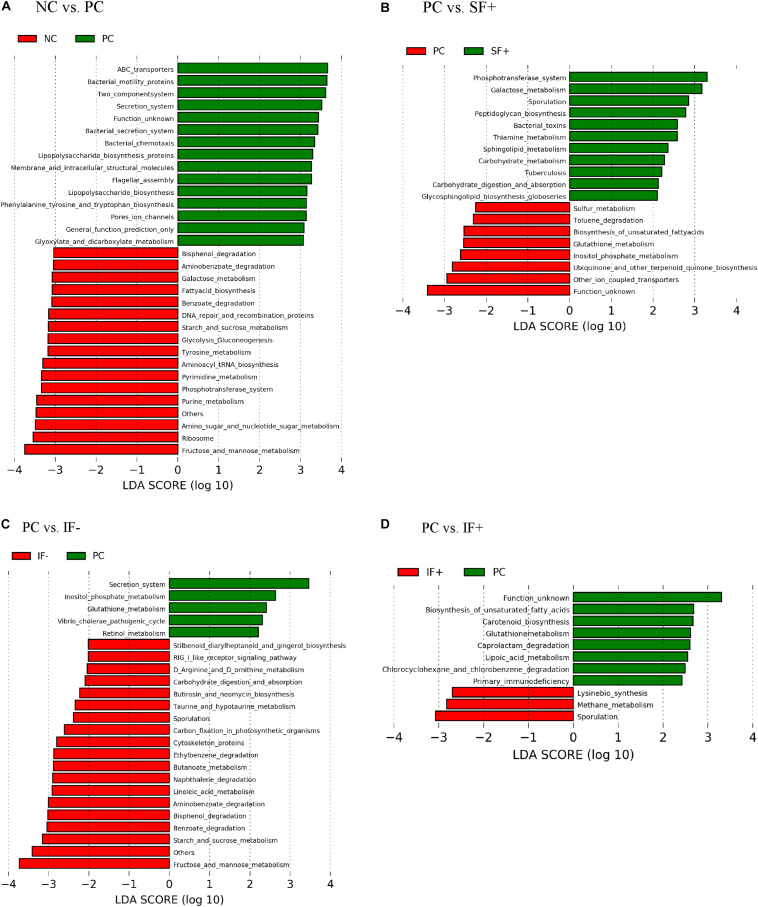
Predicted functional metagenomes of ileal digesta microbiota of pigs fed **(A)** control diet without ETEC (NC) and with ETEC (PC); **(B)** PC and soluble fiber diet with carbohydrases (SF+); **(C)** PC and insoluble fiber diet without carbohydrases (IF-); **(D)** PC and insoluble fiber diet with enzymes (IF+). Linear discriminant analysis (LDA) was performed to identify significant changes in the proportion of reconstructed functional pathways obtained from PICRUSt predictive algorithms at Kyoto Encyclopedia of Genes and Genomes (KEGG). Analysis was performed using linear discriminant analysis of effect size (LEfSe).

As illustrated in Figure 6A, various functional pathways were overrepresented (LDA score > 3) in the ileal microbiota of pigs fed PC, such as ABC transporters, bacterial motility proteins, secretion system, lipopolysaccharide biosynthesis, and flagella assembly. Ileal microbiota of pigs on NC had enriched normal carbohydrate and nucleotide metabolism, such as galactose metabolism, starch and sucrose metabolism, phosphotransferase system, and pyrimidine and purine metabolism. Figure 6B depicts that enriched functional pathways in the microbiota of pigs fed SF+ resembled those in NC, such as galactose metabolism and phosphotransferase system. Additionally, peptidoglycan biosynthesis, carbohydrate metabolism, and carbohydrate digestion and absorption were overrepresented in the ileal microbiota of pigs fed SF+. By contrast, functional pathways enriched in the ileal microbiota of pigs on PC were sulfur and glutathione metabolism. Fructose and mannose metabolism, starch and sucrose metabolism, benzoate degradation, bisphenol degradation, and aminobenzoate degradation were enriched (LDA score > 3) in the ileal microbiota of pigs fed IF- compared to PC pigs (Figure 6C). The ileal microbiota of pigs fed IF + had enriched sporulation and methane metabolism compared to those fed PC (Figure 6D). Functional pathways enriched in colonic microbiota are shown in Supplementary Figure S3.

### Correlation Between VFA and Microbiota of the Colon Digesta

**FIGURE 7:**
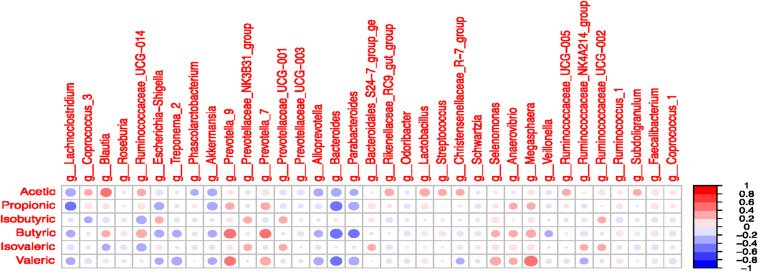
Correlation coefficients between volatile fatty acids and most abundant microbial taxa (>1% in at least one treatment) in the colonic digesta.

Significant correlations were observed between selected taxa and VFA in the colon digesta (Figure 7 and Supplementary Table S13). A positive correlation was observed between *Blautia* and acetate (*R* = 0.41; *P* < 0.05). The *Lachnoclostridium* was negatively correlated with propionate (*R* = -0.41; *P* < 0.05). The presence of *Prevotella* 7 and *Prevotella* 9 was positively correlated with butyrate and valerate (*P* < 0.05). The *Bacteroides* was negatively correlated with propionate, butyrate, and valerate (*P* < 0.05). The *Parabacteroides* was negatively correlated with butyrate (*R* = -0.41; *P* < 0.05). The presence of *Selenomonas* and *Megasphaera* was positively correlated with valerate (*P* < 0.05). The *Ruminococcaceae UCG-*014 tended to be positively correlated with butyrate and negatively correlated with isobutyrate (*P* < 0.10).

### Intestinal VFA and pH

Pigs on the NC treatment had greater amount of total VFA (*P* < 0.05) in both the cecum and colon and tended (*P* < 0.10) to have greater concentrations of acetate and propionate in the colon than those on PC (Table 1). Cecal propionate and colonic valerate and isovalerate were lower (*P* < 0.05) in pigs on PC than those on NC. The SF+ increased cecal acetate compared to PC (*P* < 0.05). In the colon, SF increased acetate concentration compared to PC, regardless of carbohydrase addition (*P* < 0.05). However, greater propionate, butyrate, total VFA, and lower isobutyrate in the colon, were only observed in pigs fed SF- compared to those fed PC (*P* < 0.05). The SF+ tended to increase total VFA compared to PC (*P* < 0.10). Cecal digesta pH did not differ among treatments. Colonic pH tended (*P* < 0.10) to be lower in SF- and was significantly lower (*P* < 0.05) in pigs fed SF+ than those fed PC. Insoluble fiber, irrespective of enzyme addition, had no impact on any VFA, but tended to decrease pH in the colon compared to PC (*P* < 0.10). In both cecum and colon, the main effect of SF increased acetate and total VFA compared with IF (*P* < 0.05). The main effect of enzyme supplementation decreased cecal valerate and colonic propionate and valerate compared to diets without enzymes (*P* < 0.05).

**TABLE 1:** Effect of soluble or insoluble fiber diet without or with carbohydrases on intestinal volatile fatty acids (VFA) in weaned pigs challenged with ETEC F18^a^.

Item	Treatment^b^							SEM	Contrast *P*-value^c^			
	
	NC	PC	SF		IF			Fiber	Enzyme	F × E
				
			SF−	SF+	IF−	IF+				
**Cecal VFA, μmol/g digesta**										
Acetate	67.6	54.8	67.1	76.2*	44.0	40.0	5.4	<0.001	0.214	0.713
Propionate	42.6*	30.5	31.6	32.0	25.8	25.0	3.3	0.061	0.940	0.853
Butyrate	18.5	13.6	16.4	14.8	13.0	11.8	2.6	0.228	0.595	0.932
Valerate	3.7^#^	2.2	2.5	0.9	2.0	1.8	0.6	0.758	0.033	0.580
Total	132.4*	101.0	117.6	123.8	84.8	86.8	10.2	0.002	0.694	0.839
pH	5.59	5.55	5.41	5.18	5.44	5.58	0.24	0.219	0.785	0.282
**Colonic VFA, μmol/g digesta**										
Acetate	62.2^#^	53.5	66.7*	64.9*	59.1	53.4	3.1	0.005	0.240	0.543
Propionate	22.8^#^	16.9	23.0*	18.6	20.4	16.6	2.0	0.256	0.046	0.870
Butyrate	12.5	9.5	15.4*	12.5	11.0	9.8	1.6	0.021	0.169	0.542
Valerate	4.2*	3.0	4.1^#^	3.1	3.6	2.8	0.4	0.349	0.025	0.831
Isobutyrate	2.0	1.8	0.7*	1.4	1.1	1.3	0.3	0.711	0.164	0.443
Isovalerate	3.6*	2.5	1.5^#^	2.3	2.0	2.3	0.4	0.524	0.152	0.414
Total	107.3*	87.1	111.7*	102.8^#^	97.3	86.2	5.9	0.014	0.106	0.858
pH	5.96	6.22	5.93^#^	5.64*	5.95^#^	5.91^#^	0.16	0.212	0.154	0.270

*Indicate significant difference compared to PC (P ≤ 0.05). ^#^Indicate tendency for significant difference compared to PC (0.05 < P ≤ 0.10). ^*a*^n = 10 pigs per treatment. ^*b*^NC, non-challenged control diet; PC, ETEC-challenged control diet; SF, soluble fiber diets without or with carbohydrases; IF, insoluble fiber diets without or with carbohydrases; SF-, soluble fiber diet without carbohydrases; SF+, soluble fiber diet with carbohydrases; IF-, insoluble fiber diet without carbohydrases; IF+, insoluble fiber diet with carbohydrases. ^c^Fiber, dietary fiber type effect (SF vs. IF); Enzyme, carbohydrase effect (without vs. with); F × E, fiber type by carbohydrase interaction effect.

## Discussion

Pathogenic challenges compromise intestinal function of pigs via different mechanisms, with one being disruption of gut microbial homestasis (Burrough et al., 2017; Argüello et al., 2018). Our previous findings generated from the same pigs used in this study revealed that an ETEC challenge resulted in increased incidence of diarrhea (40.0 vs. 7.1%) and decreased growth performance (0.13 vs. 0.22 kg/d ADG) compared to the NC; however, compared to the PC, pigs fed SF+ diets had improved growth (0.25 kg/d ADG) and pigs fed IF- diets had an increased incidence of diarrhea (57.1 vs. 40.0%, respectively) and hemolytic *E. coli* shedding (Li et al., 2019 and Supplementary Table S14). To better understand the mechanisms through which different sources of dietary fiber and exogenous carbohydrases alter the pig’s response to ETEC, this study evaluated the impact of an ETEC F18 challenge as well as potential protective effects of the diet on intestinal microbiota composition and microbial fermentation products.

Diets did not impart statistically significant differences in alpha- and beta-diversity of the fecal microbiota prior to ETEC challenge as only a few differentially abundant bacterial taxa were identified on day 7 in fecal bacteria of pigs fed different diets. The lack of detectable alteration in microbial structure and alteration in bacterial abundance is probably due to the short adaptation time (7 days) to the diets in this study. Similarly, alpha-diversity of the fecal samples was not affected by pre-feeding with dietary fiber and enzyme supplementation following the experimental challenge with ETEC. Partly due to intragroup variation in response to bacterial challenge, ETEC inoculation only moderately altered ileal microbial structure and the SF+ and IF- slightly modulated the microbial community structure compared to PC.

The ileal and colonic microbiota of pigs on NC were dominated by *Firmicutes*, consistent with previous research evaluating intestinal bacterial diversity in swine (Zhang et al., 2018). As expected, the ETEC challenge increased the relative abundance of *Proteobacteria* in both ileal and colonic digesta compared to NC; this was driven primarily by the increase in *Escherichia-Shigella*. This also agrees with attachment of *E. coli* to ileal epithelial cells, elevated fecal hemolytic *E. coli* shedding and increased incidence of diarrhea observed in these animals as described in our recent publication (Li et al., 2019). Accordingly, pigs receiving the ETEC inoculum in PC presented lower abundance of phyla *Firmicutes* and genus *Lactobacillus* than those receiving the sham inoculum, indicating disruption of microbial homeostasis by ETEC. The decreased concentration of propionate, valerate, and total VFA in the large intestine of pigs in PC in comparison to NC suggests that the ETEC challenge also impaired intestinal microbial fermentation capacity.

Pollock et al. (2018) evaluated the effect of subclinical ETEC exposure via feed on fecal microbiota in weaned pigs. Unlike what observed in the current study, the authors did not report significant changes in fecal *Enterobacteriaceae* or in microbial community structure. This implies – not surprisingly – that the dose and method of an ETEC challenge influence microbial response to the infection in pigs (Luise et al., 2019).

It has been suggested that soluble fiber favors pathogenic *E. coli* proliferation in the gut through increasing intestinal digesta viscosity (Hopwood et al., 2004; Montagne et al., 2004). However, *Escherichia-Shigella* in both ileal and colonic digesta of pigs fed SF- and PC were not different. Furthermore, the SF- decreased *E. coli* attachment in the ileum compared to PC (Li et al., 2019), indicating the beneficial effect of SF- on reducing mucosal pathogens. The SF+ tended to decrease ileal *Escherichia-Shigella* compared to PC and significantly decreased ileal *Escherichia-Shigella* compared to SF-. One potential explanation is that carbohydrases break down the SBP to release pectic oligosaccharides, exerting prebiotic effects (Leijdekkers et al., 2014).

Additionally, Högberg and Lindberg (2004) suggested that enzyme (xylanase and ß-glucananse) supplementation increased substrate for the growth of lactic acid-producing bacteria (e.g., *Lactobacillus*) in the ileum of weaning pigs. Because no significant increase in the abundance of *Lactobacillu* by SF+ was observed, other bacteria producing lactic acid may be involved to reduce *Escherichia-Shigella*. Although some *Streptococcus* is normally associated with pathogenic bacteria, some strains, such as *S. infantarius* and *S. thermophilus*, are lactic acid producers and are considered probiotics (Anadón et al., 2006; Yang et al., 2015). Therefore, the elevated abundance of *Streptococcus* in pigs fed SF+ may contribute to the reduction in *Escherichia-Shigella*. Moreover, greater abundance of *Streptococcus* was reported in IgG supplemented pigs after an ETEC F4 challenge, which was accompanied by reduced ETEC shedding (Hedegaard et al., 2016). Collectively, the observed increase in *Streptococcus* associated with feeding soluble fiber, especially SF+, points to prebiotic effects of soluble fiber after an ETEC challenge.

While *Erysipelotrichaceae* is more associated with metabolic disorder in humans (review by Kaakoush, 2015), more abundant colonic *Erysipelotrichaceae* along with *Lactobacillus* and *Roseburia* was reported in pigs that are resistant to the development of swine dysentery after experimental inoculation with *Brachyspira* (Burrough et al., 2017). This may suggest that the greater abundance of *Roseburia*, *Turicibacter*, and *Erysipelotrichaceae* in the ileum of pigs fed SF, regardless of carbohydrase addition, is in some way beneficial for defense against the ETEC infection or is a potential biomarker for a resistant phenotype. The increased abundance of *Turicibacter* and *Clostridium* in the colon of pigs fed a low-dose *Bacillus* probiotic mix during ETEC F4 infection supports this speculation (Zhang et al., 2017). Vital et al. (2014) also reported the existence of terminal *but* and *buk* genes for butyrate production in *Erysipelotrichaceae*, in agreement with the greater butyrate concentration in SF-. Additionally, increased colonic *Prevotella 7* was observed in SF- pigs and enriched colonic *Prevotella 1 and Prevotella 9* were observed in SF+ pigs; this agrees with Tian et al. (2017) who reported that pectin enriched diets increased *Prevotella*. *Prevotella 7* was positively correlated with butyrate, in line with the greater butyrate concentration in SF+ pigs. Furthermore, *Prevotella* was reported to be positively correlated with luminal secretory IgA concentrations and body weight of pigs (Mach et al., 2015). Secretory IgA has been considered as a first line of defense in protecting the intestinal epithelium from enteric pathogens and toxins (Mantis et al., 2011). Therefore, similar alterations in ileal microbiota of pigs in this study may contribute to previously observed improvement in growth in SF+ (Li et al., 2019).

Pigs fed IF- tended to decrease *Enterobacteriaceae* compared to PC, which concurred with a numerical increase in *Lactobacillus.* This seems to contradict previous findings that IF- increased fecal hemolytic *E. coli* shedding and the incidence of diarrhea (Li et al., 2019). The reason for this is unclear; it is likely that the consumption of IF reduces digesta transit time in the intestine (Wilfart et al., 2007), which then increases the clearance of pathogenic bacteria, resulting in increased hemolytic *E. coli* shedding. This can explain the tendency for greater hemolytic *E. coli* shedding in IF+ than PC, along with increases in beneficial bacteria, including *Lactobacillus*, *Lachnospiraceae*, and *Roseburia*. However, increased diarrhea in IF- relative to PC suggests more ETEC attachment in the intestine. Therefore, these data indicate that the alteration of the intestinal microbial community structure and the complex interplay within the intestinal community may play a more important role than simple changes in the abundance of individual or a few microbial species in determining responses of pigs to an ETEC infection.

Pigs fed SF- and SF+ appeared to present an improved fermentation capacity than those fed PC, as indicated by the greater colonic VFA concentration. Compelling evidence suggests that VFA, especially acetate and butyrate, can improve gut barrier function and protect the host against bacterial infections (Peng et al., 2009; Fukuda et al., 2011). Moreover, decreased colonic pH by SF- and SF+ may be beneficial to pigs because low pH can inhibit the growth of pathogenic bacteria (Suiryanrayna and Ramana, 2015). Exogenous enzyme supplementation, irrespective of dietary fiber source, reduced the concentration of cecal valerate and colonic propionate and valerate compared to diets without enzymes. In agreement with the current VFA data, Clarke et al. (2018) and Li et al. (2018) also reported reduced total VFA in the large intestine by supplementing enzymes. These results support the view of Bedford and Cowieson (2012) who suggest that carbohydrase enzyme addition shift the degradation of fiber from the hindgut to the upper gut.

To further characterize the functional profiling of microbiota, predictive functional analysis was also performed. These analyses were based on reassigning OTU taxa labels using Greengenes 13.5, which is the database used by PICRUST. Greengenes 13.5 is considerably older than Silva 128, which was used elsewhere throughout this work and may affect the predictions. The agreement in taxa assignments are good through the family level (adjusted Rand indices: 0.95 at phylum, 0.80 at class, 0.77 at order, 0.69 family, but only 0.08 at the genus level). The disagreement in genus names is mainly attributed by unclassified genus with Greengenes 13.5.

The predictive functional analysis revealed that the bacterial functional pathways, including ABC transporters, bacterial motility proteins, lipopolysaccharide biosynthesis, and glutathione metabolism, were enriched in the ileal microbiota of PC pigs. Bacterial pathogens can invade mammalian hosts, damage tissue, and elicit innate immune system through a multitude of methods, with the secretion of proteins across phospholipid membranes being an essential component of those strategies (Green and Mecsas, 2016). ABC transporter complex LptBFG is involved in the translocation of lipopolysaccharides from the inner to the outer membrane of Gram-negative bacteria (Narita and Tokuda, 2009). Glutathione metabolism is also found primarily in Gram-negative bacteria (Smirnova and Oktyabrsky, 2005). Thus, the enriched KEGG functional pathways of the ileal microbiota in pigs on PC may indicate enhanced proliferation and metabolism of Gram-negative pathogenic bacteria; this hypothesis is in accordance with the increased abundance of *Escherichia-Shigella*. The functional pathway involving sulfur metabolism was also enriched in the microbiota of the ileal digesta of pigs fed the PC compared to SF+. It may suggest increased microbial degradation of sulfated compounds, such as mucins and sulfur-containing amino acids; this process produces toxic hydrogen sulfide that may damage the intestinal mucosal barrier (Carbonero et al., 2012). Conversely, enriched functional pathways in the ileal microbiota of pigs fed SF+ resembled those fed NC, such as phosphotransferase system and galactose metabolism related to bacterial sugar transport and metabolism (Deutscher et al., 2006); this probably suggests better microbial homeostasis compared with pigs on PC.

## Conclusion

In conclusion, an ETEC challenge disrupted gut microbial homeostasis by increasing *Escherichia-Shigella* and decreasing *Lactobacillus*. The ETEC infection compromised microbial fermentation capacity as shown by the reduced total VFA. The inclusion of SF or IF without or with carbohydrases altered intestinal microbiota in different ways to maintain or restore microbial homeostasis. The pigs fed SF+ presented increased cecal acetate and reduced colonic pH, which may be beneficial for intestinal health. Taken together, in combination with results from Li et al. (2019), these data suggest that the inclusion of a soluble and highly fermentable fiber from SBP with carbohydrase supplementation may help protect pigs against moderate ETEC infection.

## Data Availability Statement

The datasets generated for this study can be found in the European Nucleotide Archive (ENA), PRJEB36303, ERP119474.

## Ethics Statement

All procedures in this experiment were reviewed and approved by the Institutional Animal Care and Use Committee at Iowa State University (IACUC #6-16-8306-S and #16-I-0027-A).

## Author Contributions

QL, EB, NG, and CL designed the experiment. QL performed the experiment and analyzed data with assistance of SG in the animal trial. OS donated and prepared the ETEC F18 inoculum. XP performed the bioinformatic analysis of the 16S rRNA sequencing data with assistance of KD. EB contributed to the microbial sequencing data analysis and results interpretation. JP was the principal investigator who supervised all aspects of the study. All the authors reviewed and approved the final manuscript.

## Conflict of Interest

The authors declare that the research was conducted in the absence of any commercial or financial relationships that could be construed as a potential conflict of interest.
