# Supplementary material for: Dietary Soluble and Insoluble Fiber With or Without Enzymes Altered the Intestinal Microbiota in Weaned Pigs Challenged With Enterotoxigenic E. coli F18
Source: Front Microbiol. 2020 May 27;11:1110. doi: 10.3389/fmicb.2020.01110 (PMC7267687; doi:10.3389/fmicb.2020.01110)
Supplement: Supplementary file 1 [file Data_Sheet_1.docx]

Supplementary Material

**Supplemental Tables**

**Table S1.** Ingredient composition of the experimental diets (as-fed basis, %)

| Item | Diets^1^ | | |
| --- | --- | --- | --- |
|  | Control | SF | IF |
| Corn | 41.79 | 42.11 | 42.40 |
| Cornstarch | 15.00 | 5.00 | - |
| Soybean meal, 46.5% CP | 15.00 | 15.00 | 15.00 |
| DDGS^2^ | - | - | 15.00 |
| Sugar beet pulp | - | 10.00 | - |
| Fish meal, menhaden select | 5.00 | 5.00 | 5.00 |
| Whey powder, > 61% lactose | 13.50 | 13.50 | 13.50 |
| Casein | 4.00 | 4.00 | 4.00 |
| Soybean oil | 2.50 | 2.50 | 2.50 |
| Monocalcium phosphate | 0.56 | 0.56 | 0.20 |
| Limestone | 0.95 | 0.73 | 1.10 |
| Sodium chloride | 0.25 | 0.25 | 0.25 |
| L-Lys HCl | 0.50 | 0.46 | 0.42 |
| DL-Met | 0.23 | 0.22 | 0.11 |
| L-Thr | 0.20 | 0.18 | 0.09 |
| L-Trp | 0.05 | 0.04 | 0.03 |
| L-Val | 0.07 | 0.05 | - |
| Vitamin premix^3^ | 0.25 | 0.25 | 0.25 |
| Trace mineral premix^4^ | 0.15 | 0.15 | 0.15 |
| **Calculated nutrient levels, %** |  |  |  |
| ME, Mcal/kg | 3.53 | 3.43 | 3.42 |
| NE, Mcal/kg | 2.68 | 2.53 | 2.50 |
| Crude protein | 19.96 | 20.71 | 23.67 |
| Ether extract | 4.67 | 4.77 | 5.22 |
| Neutral detergent fiber | 5.04 | 9.56 | 10.16 |
| Acid detergent fiber | 2.00 | 4.35 | 4.55 |
| Total dietary fiber | 8.24 | 14.99 | 13.03 |
| Starch | 40.07 | 31.17 | 28.30 |
| Calcium | 0.80 | 0.80 | 0.80 |
| Total P | 0.60 | 0.61 | 0.64 |
| STTD P | 0.43 | 0.43 | 0.43 |
| SID Lys | 1.44 | 1.44 | 1.44 |
| SID Met + Cys | 0.79 | 0.79 | 0.79 |
| SID Thr | 0.85 | 0.85 | 0.85 |
| SID Trp | 0.26 | 0.26 | 0.26 |

^1^SF: soluble fiber diets without or with carbohydrases; IF: insoluble fiber diets without or with carbohydrases; the carbohydrases contained 0.01% pectinase (Pectinase ABE; 56 PE per kg of diet), 0.01% xylanase (Econase XT; 19,000 BXU per kg of diet), and 0.001% β-glucanase (Econase GT P; 23,200 BU per kg of diet), and the supplementation rate was based on the manufacturer’s recommendations (AB Vista, Plantation, FL).

^2^Distiller’s dried grains with solubles (DDGS).

^3^Provided per kg of diet: 7,656 IU vitamin A, 875 IU vitamin D, 63 IU vitamin E, 4 mg vitamin K, 70 mg niacin, 34 mg pantothenic acid, 14 mg riboflavin, and 0.06 mg vitamin B_12._

^4^Provided per kg of diet: 165 mg Zn (zinc sulfate), 165 mg Fe (iron sulfate), 39 mg Mn (manganese sulfate), 17 mg Cu (copper sulfate), 0.3 mg I (calcium iodate), and 0.3 mg Se (sodium selenite).

**Table S2.** Analyzed nutrient composition of fibrous ingredients and experimental diets (as-fed basis, %)

| Item^1^ | Ingredient^2^ | |  | Diets^3^ | | | | |
| --- | --- | --- | --- | --- | --- | --- | --- | --- |
|  | Beet pulp | DDGS |  | Control | SF- | SF+ | IF- | IF+ |
| Dry matter | 92.78 | 89.57 |  | 89.77 | 90.05 | 90.06 | 89.98 | 89.63 |
| GE, Mcal/kg | 3.82 | 4.48 |  | 4.02 | 4.05 | 4.06 | 4.17 | 4.17 |
| Crude protein | 9.41 | 28.09 |  | 18.40 | 19.38 | 19.74 | 23.21 | 22.32 |
| aEE | 1.94 | 7.04 |  | 5.00 | 5.33 | 5.57 | 6.61 | 6.25 |
| NDF | 35.70 | 28.67 |  | 4.83 | 8.52 | 8.32 | 9.54 | 9.67 |
| ADF | 22.14 | 7.85 |  | 1.48 | 3.44 | 3.22 | 2.65 | 2.51 |
| Hemicellulose | 13.56 | 20.83 |  | 3.34 | 5.08 | 5.10 | 6.88 | 7.16 |
| SDF | 17.10 | 1.70 |  | 0.70 | 2.10 | 1.90 | 1.00 | 1.20 |
| IDF | 43.80 | 31.10 |  | 8.60 | 11.60 | 11.20 | 11.30 | 11.40 |
| TDF | 60.90 | 32.80 |  | 9.30 | 13.70 | 13.10 | 12.30 | 12.60 |

^1^GE: gross energy; aEE: acid ether extract; NDF: neutral detergent fiber; ADF: acid detergent fiber; SDF: soluble dietary fiber; IDF: insoluble dietary fiber; TDF: total dietary fiber.

^2^Distillers dried grains with solubles.

^3^SF-: soluble fiber diet without carbohydrases; SF+: soluble fiber diet with carbohydrases; IF-: insoluble fiber diet without carbohydrases; IF+: insoluble fiber diet with carbohydrases; the carbohydrases contained 0.01% pectinase (Pectinase ABE; 56 PE per kg of diet), 0.01% xylanase (Econase XT; 19,000 BXU per kg of diet), and 0.001% β-glucanase (Econase GT P; 23,200 BU per kg of diet), and the supplementation rate was based on the manufacturer’s recommendations (AB Vista, Plantation, FL).

**Table S3.** Relative abundance of bacterial phylum in **feces** of pigs fed basal control diets, soluble fiber diets without (SF-) or with enzymes (SF+), or insoluble fiber diets without (IF-) or with enzymes (IF+), %^1^

| Item | Treatment | | | | | | | *P*-value | |
| --- | --- | --- | --- | --- | --- | --- | --- | --- | --- |
|  | Basal |  | SF | |  | IF | | SF- vs. SF+ | IF- vs. IF+ |
|  |  |  | SF- | SF+ |  | IF- | IF+ |  |  |
| *Actinobacteria* | 1.90 |  | 1.70 | 1.72 |  | 1.58 | 2.03 | NS | NS |
| *Bacteroidetes* | 24.55 |  | 28.52 | 28.01 |  | 28.67 | 32.44 | NS | NS |
| *Firmicutes* | 65.62 |  | 62.94 | 63.06 |  | 62.49 | 55.35 | NS | NS |
| *Fusobacteria* | 0.38 |  | 0.06 | 1.01 |  | 0.07 | 0.04 | NS | NS |
| *Proteobacteria* | 1.63 |  | 0.93 | 0.97 |  | 1.13 | 1.18 | NS | NS |
| *Saccharibacteria* | 3.39 |  | 4.89 | 4.10 |  | 3.98 | 8.17 | NS | NS |
| *Spirochaetae* | 0.75 |  | 0.04 | 0.03 |  | 0.05 | 0.10 | NS | NS |
| *Tenericutes* | 0.93 |  | 0.36 | 0.44 |  | 0.62 | 0.44 | NS | NS |
| *Verrucomicrobia* | 0.61 |  | 0.04 | 0.06 |  | 0.01 | 0.01 | NS | NS |

^1^n = 6 pigs per treatment except for basal with 13 pigs and SF+ with 8 pigs.

**Table S4.** Relative abundance of bacterial family in **feces** of pigs fed basal control diets, soluble fiber diets without (SF-) or with enzymes (SF+), or insoluble fiber diets without (IF-) or with enzymes (IF+), %^1^

| Item | Treatment | | | | | | |
| --- | --- | --- | --- | --- | --- | --- | --- |
|  | Basal |  | SF | |  | IF | |
|  |  |  | SF- | SF+ |  | IF- | IF+ |
| ***Actinobacteria*** |  |  |  |  |  |  |  |
| *Coriobacteriaceae* | 1.12 |  | 1.11 | 1.05 |  | 1.48 | 1.58 |
| ***Bacteroidetes*** |  |  |  |  |  |  |  |
| *Bacteroidaceae* | 1.20 |  | 0.15 | 0.32 |  | 0.14 | 0.29 |
| *Bacteroidales S24-7 group* | 4.37 |  | 5.87 | 8.03 |  | 5.98 | 6.30 |
| *Prevotellaceae* | 14.51 |  | 15.71 | 16.10 |  | 17.28 | 20.64 |
| *Rikenellaceae* | 2.50 |  | 4.45 | 2.19 |  | 4.32 | 3.58 |
| ***Firmicutes*** |  |  |  |  |  |  |  |
| *Acidaminococcaceae* | 9.89 |  | 14.84 | 12.05 |  | 10.65 | 10.27 |
| *Christensenellaceae* | 2.11 |  | 1.21 | 2.61 |  | 2.27 | 1.32 |
| *Clostridiaceae 1* | 0.15 |  | 0.08 | 0.16 |  | 1.21 | 0.16 |
| *Lachnospiraceae* | 6.07 |  | 5.74 | 6.72 |  | 6.81 | 4.73 |
| *Lactobacillaceae* | 7.38 |  | 4.01 | 5.57 |  | 3.93 | 2.48 |
| *Ruminococcaceae* | 18.13 |  | 20.39 | 17.14 |  | 18.40 | 16.09 |
| *Streptococcaceae* | 0.09 |  | 0.16 | 1.04 |  | 0.29 | 0.08 |
| *Veillonellaceae* | 17.10 |  | 12.02 | 11.10 |  | 15.46 | 11.01 |
| ***Planctomycetes*** |  |  |  |  |  |  |  |
| *Planctomycetaceae* | 0.38 |  | 0.06 | 1.01 |  | 0.07 | 0.04 |
| ***Spirochaetae*** |  |  |  |  |  |  |  |
| *Spirochaetaceae* | 3.39 |  | 4.89 | 4.10 |  | 3.98 | 8.17 |

^1^n = 10 pigs per treatment except for basal with 13 pigs and SF+ with 8 pigs.

**Table S5.** Relative abundance of bacterial genus in **feces** of pigs fed basal control diets, soluble fiber diets without (SF-) or with enzymes (SF+), or insoluble fiber diets without (IF-) or with enzymes (IF+), %^1^

| Taxonomy | Item | Treatment | | | | | | |
| --- | --- | --- | --- | --- | --- | --- | --- | --- |
|  |  | Basal |  | SF | |  | IF | |
|  |  |  |  | SF- | SF+ |  | IF- | IF+ |
| ***Bacteroidetes*** |  |  |  |  |  |  |  |  |
| *Bacteroidaceae* | *Bacteroides* | 1.20 |  | 0.15 | 0.32 |  | 0.14 | 0.29 |
| *Bacteroidales S24-7 group* | *Bacteroidales S24-7 group ge* | 4.37 |  | 5.87 | 8.03 |  | 5.98 | 6.30 |
| *Prevotellaceae* | *Alloprevotella* | 2.61 |  | 1.65 | 2.69 |  | 3.53 | 1.68 |
| *Prevotellaceae* | *Prevotella 1* | 0.86 |  | 1.55 | 0.23 |  | 0.51 | 2.45 |
| *Prevotellaceae* | *Prevotella 7* | 0.21 |  | 0.32 | 1.71 |  | 0.01 | 0.05 |
| *Prevotellaceae* | *Prevotella 9* | 0.93 |  | 1.88 | 4.13 |  | 1.46 | 2.99 |
| *Prevotellaceae* | *Prevotellaceae NK3B31 group* | 1.78 |  | 2.31 | 2.21 |  | 1.69 | 3.68 |
| *Prevotellaceae* | *Prevotellaceae UCG-001* | 1.36 |  | 0.23 | 0.34 |  | 1.17 | 2.25 |
| *Prevotellaceae* | *Prevotellaceae UCG-003* | 0.66 |  | 0.76 | 0.43 |  | 3.04 | 0.52 |
| *Prevotellaceae* | *uncultured* | 0.05 |  | 0.01 | 0.21 |  | 0.30 | 1.70 |
| *Rikenellaceae* | *Rikenellaceae RC9 gut group* | 2.18 |  | 4.36 | 2.10 |  | 4.11 | 3.42 |
| ***Firmicutes*** |  |  |  |  |  |  |  |  |
| *Acidaminococcaceae* | *Phascolarctobacterium* | 9.48 |  | 14.57 | 11.62 |  | 10.40 | 10.05 |
| *Christensenellaceae* | *Christensenellaceae R-7 group* | 2.08 |  | 1.18 | 2.58 |  | 2.26 | 1.30 |
| *Clostridiaceae 1* | *Clostridium sensu stricto 1* | 0.11 |  | 0.06 | 0.15 |  | 1.11 | 0.11 |
| *Lachnospiraceae* | *Lachnospiraceae AC2044 group* | 1.05 |  | 0.53 | 0.15 |  | 0.69 | 0.25 |
| *Lachnospiraceae* | *Lachnospiraceae NK4A136 group* | 0.51 |  | 0.31 | 0.53 |  | 0.43 | 1.17 |
| *Lachnospiraceae* | *Lachnospiraceae XPB1014 group* | 0.37 |  | 0.66 | 1.26 |  | 0.40 | 0.39 |
| *Lactobacillaceae* | *Lactobacillus* | 7.38 |  | 4.01 | 5.57 |  | 3.93 | 2.48 |
| *Ruminococcaceae* | *Faecalibacterium* | 2.32 |  | 0.25 | 0.51 |  | 0.75 | 0.43 |
| *Ruminococcaceae* | *Oscillospira* | 0.43 |  | 1.15 | 0.28 |  | 0.46 | 0.32 |
| *Ruminococcaceae* | *Ruminococcaceae ge* | 1.00 |  | 1.51 | 1.26 |  | 1.75 | 0.76 |
| *Ruminococcaceae* | *Ruminococcaceae NK4A214 group* | 1.49 |  | 1.72 | 1.54 |  | 1.69 | 1.63 |
| *Ruminococcaceae* | *Ruminococcaceae UCG-002* | 3.52 |  | 3.31 | 4.06 |  | 3.04 | 4.39 |
| *Ruminococcaceae* | *Ruminococcaceae UCG-005* | 1.31 |  | 1.81 | 2.43 |  | 2.45 | 1.00 |
| *Ruminococcaceae* | *Ruminococcus 1* | 0.89 |  | 3.27 | 1.44 |  | 1.57 | 1.97 |
| *Streptococcaceae* | *Streptococcus* | 0.09 |  | 0.16 | 1.04 |  | 0.29 | 0.08 |
| *Veillonellaceae* | *Anaerovibrio* | 0.88 |  | 1.55 | 2.09 |  | 0.91 | 1.33 |
| *Veillonellaceae* | *Megasphaera* | 14.10 |  | 9.60 | 7.72 |  | 11.38 | 6.90 |
| ***Spirochaetae*** |  |  |  |  |  |  |  |  |
| *Spirochaetaceae* | *Treponema 2* | 3.36 |  | 4.85 | 4.08 |  | 3.94 | 8.11 |

^1^n = 10 pigs per treatment except for basal with 13 pigs and SF+ with 8 pigs.

**Table S6.** Differential abundance in specific taxa in **feces** of pigs fed basal control diets, soluble fiber diets without (SF-) or with enzymes (SF+), or insoluble fiber diets without (IF-) or with enzymes (IF+) according to DESeq2

| Treatments compared | BaseMean^1^ | Log2(fold change) | lfcSE^2^ | padj^3^ | Taxonomic annotation |
| --- | --- | --- | --- | --- | --- |
| SF- over Basal control | 78.17 | -3.54 | 1.10 | 0.018 | *Bacteroidaceae* |
|  | 117.68 | -3.92 | 1.14 | 0.011 | *Bacteroides* |
|  | 275.90 | -4.12 | 1.25 | 0.016 | *Faecalibacterium* |
| SF+ over Basal control | 1471.90 | 3.16 | 1.04 | 0.029 | *Streptococcaceae* |
| IF- over Basal control | 78.17 | -3.61 | 1.20 | 0.032 | *Bacteroidaceae* |
|  | 117.68 | -4.41 | 1.25 | 0.010 | *Bacteroides* |
| IF+ over Basal control | 49574.35 | -2.47 | 0.84 | 0.046 | *Lactobacillaceae* |
|  | 60.84 | 4.50 | 1.41 | 0.044 | *uncultured* |

^1^Mean of normalized counts for all samples.

^2^Standard error of DESeq2.

^3^padj is the Benjamini–Hochberg adjusted *P* value.

**Table S7.** Relative abundance of bacterial taxa in **ileal digesta** of pigs fed control diet without ETEC (NC), control diet with ETEC (PC), soluble fiber diet without (SF-) or with enzymes (SF+), or insoluble fiber diet without (IF-) or with enzymes (IF+) after an ETEC F18 challenge, %^1^

| Item | Treatment | | | | | | | |
| --- | --- | --- | --- | --- | --- | --- | --- | --- |
|  | NC | PC |  | SF | |  | IF | |
|  |  |  |  | SF- | SF+ |  | IF- | IF+ |
| ***Actinobacteria*** | 0.93 | 0.56 |  | 0.44 | 0.79^#^ |  | 0.77 | 0.36 |
| ***Bacteroidetes*** | 0.17 | 1.12 |  | 0.42^#^ | 1.28^#^ |  | 0.05 | 1.02 |
| *Prevotellaceae* | 0.07 | 0.24 |  | 0.39 | 1.19 |  | 0.04 | 0.88 |
| ***Firmicutes*** | 97.40^*^ | 47.28 |  | 59.96 | 90.25 |  | 91.93 | 76.62 |
| *Clostridiaceae 1* | 0.32 | 0.11 |  | 1.50 | 8.52 |  | 0.38 | 6.80 |
| *Clostridium sensu stricto 1* | 0.30 | 0.07 |  | 1.40 | 7.82 |  | 0.01 | 6.75 |
| *Erysipelotrichaceae* | 0.06 | 0.06 |  | 1.19 | 1.64 |  | 0.01 | 0.20 |
| *Turicibacter* | 0.05 | 0.05 |  | 1.16 | 1.62 |  | 0.00 | 0.18 |
| *Lachnospiraceae* | 0.01 | 0.03 |  | 0.03 | 0.10 |  | 0.08 | 5.37 |
| *Roseburia* | 0.002 | 0.0003 |  | 0.003 | 0.003 |  | 0.001 | 4.28 |
| *Lactobacillaceae* | 91.99 | 44.32 |  | 48.94 | 59.03 |  | 82.74 | 57.92 |
| *Lactobacillus* | 91.99 | 44.32 |  | 48.94 | 59.03 |  | 82.74 | 57.92 |
| *Streptococcaceae* | 0.64 | 0.34 |  | 4.35 | 16.38 |  | 0.31 | 0.23 |
| *Streptococcus* | 0.53 | 0.32 |  | 4.35 | 16.34 |  | 0.27 | 0.22 |
| *Veillonellaceae* | 3.79 | 2.05 |  | 2.62 | 3.52 |  | 7.79 | 5.17 |
| *Megasphaera* | 1.71 | 0.71 |  | 2.04 | 2.14 |  | 4.28 | 2.29 |
| *Veillonella* | 1.00 | 0.83 |  | 0.16 | 0.55 |  | 3.07 | 1.95 |
| ***Proteobacteria*** | 1.44^*^ | 51.01 |  | 39.16 | 7.68 |  | 7.23 | 21.98 |
| *Enterobacteriaceae* | 0.49 | 40.40 |  | 38.70 | 4.99 |  | 4.39 | 14.05 |
| *Escherichia-Shigella* | 0.47 | 40.39 |  | 38.69 | 4.97 |  | 4.38 | 13.96 |
| *Pasteurellaceae* | 0.89 | 8.91 |  | 0.25^*^ | 1.79 |  | 2.76 | 7.13 |
| *Actinobacillus* | 0.84 | 8.17 |  | 0.23 | 1.66 |  | 2.55 | 6.23 |

^*^Indicate significant difference compared to PC (*P* ≤ 0.05).

^#^Indicate tendency for significant difference compared to PC (0.05 < *P* ≤ 0.10).

^1^n = 10 pigs per treatment except for IF- with 9 pigs.

**Table S8.** Relative abundance of bacterial phylum in **colonic digesta** of pigs fed control diet without ETEC (NC), control diet with ETEC (PC), soluble fiber diet without (SF-) or with enzymes (SF+), or insoluble fiber diet without (IF-) or with enzymes (IF+) after an ETEC F18 challenge, %^1^

| Item | Treatment | | | | | | | | *P*-value | |
| --- | --- | --- | --- | --- | --- | --- | --- | --- | --- | --- |
|  | NC | PC |  | SF | |  | IF | | SF- vs. SF+ | IF- vs. IF+ |
|  |  |  |  | SF- | SF+ |  | IF- | IF+ |  |  |
| *Actinobacteria* | 1.27^#^ | 0.63 |  | 0.64 | 1.19 |  | 0.92 | 0.46 | NS | NS |
| *Bacteroidetes* | 17.91 | 27.27 |  | 26.11 | 22.62 |  | 25.95 | 31.48 | NS | NS |
| *Firmicutes* | 79.62^*^ | 56.14 |  | 63.50 | 71.83 |  | 68.49 | 60.65 | NS | NS |
| *Fusobacteria* | 0.00 | 0.77 |  | 0.18 | 0.00 |  | 0.00 | 0.01 | NS | NS |
| *Proteobacteria* | 0.27^*^ | 5.69 |  | 7.85 | 1.45 |  | 1.69 | 3.59 | NS | NS |
| *Saccharibacteria* | 0.08 | 0.01 |  | 0.03 | 0.37 |  | 0.66 | 0.05 | NS | NS |
| *Spirochaetae* | 0.47 | 1.74 |  | 1.14 | 1.42 |  | 1.73 | 3.11 | NS | NS |
| *Tenericutes* | 0.11 | 0.13 |  | 0.08 | 0.69 |  | 0.24 | 0.25 | NS | NS |
| *Verrucomicrobia* | 0.02 | 7.01 |  | 0.00 | 0.07 |  | 0.01 | 0.15 | NS | NS |

^*^Indicate significant difference compared to PC (*P* ≤ 0.05).

^#^Indicate tendency for significant difference compared to PC (0.05 < *P* ≤ 0.10).

^1^n = 10 pigs per treatment except for SF- with 8 pigs.

**Table S9.** Relative abundance of bacterial family in **colonic digesta** of pigs fed control diet without ETEC (NC), control diet with ETEC (PC), soluble fiber diet without (SF-) or with enzymes (SF+), or insoluble fiber diet without (IF-) or with enzymes (IF+) after an ETEC F18 challenge, %^1^

| Item | Treatment | | | | | | | |
| --- | --- | --- | --- | --- | --- | --- | --- | --- |
|  | NC | PC |  | SF | |  | IF | |
|  |  |  |  | SF- | SF+ |  | IF- | IF+ |
| ***Actinobacteria*** |  |  |  |  |  |  |  |  |
| *Coriobacteriaceae* | 1.10 | 0.53 |  | 0.58 | 0.88 |  | 0.75 | 0.38 |
| ***Bacteroidetes*** |  |  |  |  |  |  |  |  |
| *Bacteroidaceae* | 0.27 | 1.39 |  | 1.53 | 0.29 |  | 0.15 | 0.48 |
| *Bacteroidales S24-7 group* | 2.27 | 2.68 |  | 1.31 | 2.28 |  | 2.81 | 3.05 |
| *Porphyromonadaceae* | 0.18 | 1.65 |  | 1.27 | 0.29 |  | 0.50 | 0.52 |
| *Prevotellaceae* | 10.93 | 16.97 |  | 16.49 | 11.74 |  | 17.49 | 21.71 |
| *Rikenellaceae* | 3.51 | 3.20 |  | 4.58 | 7.54 |  | 4.51 | 4.01 |
| ***Firmicutes*** |  |  |  |  |  |  |  |  |
| *Acidaminococcaceae* | 17.60 | 14.69 |  | 16.63 | 12.22 |  | 19.35 | 19.93 |
| *Christensenellaceae* | 0.52 | 0.49 |  | 0.44 | 1.02 |  | 0.44 | 0.44 |
| *Lachnospiraceae* | 7.93 | 8.51 |  | 10.68 | 13.04 |  | 10.57 | 8.74 |
| *Lactobacillaceae* | 18.35 | 5.41 |  | 5.62 | 11.77 |  | 10.50 | 5.66 |
| *Ruminococcaceae* | 14.94 | 10.81 |  | 13.59 | 19.38 |  | 12.71 | 15.29 |
| *Streptococcaceae* | 0.03 | 0.10 |  | 0.13 | 1.06 |  | 0.09 | 0.05 |
| *Veillonellaceae* | 17.91 | 12.94 |  | 13.23 | 9.39 |  | 12.68 | 6.29 |
| ***Proteobacteria*** |  |  |  |  |  |  |  |  |
| *Enterobacteriaceae* | 0.03 | 4.22 |  | 7.03 | 0.94 |  | 0.71 | 2.33 |
| ***Spirochaetae*** |  |  |  |  |  |  |  |  |
| *Spirochaetaceae* | 0.47 | 1.74 |  | 1.14 | 1.42 |  | 1.73 | 3.11 |
| ***Verrucomicrobia*** |  |  |  |  |  |  |  |  |
| *Verrucomicrobiaceae* | 0.01 | 6.96 |  | 0.00 | 0.05 |  | 0.01 | 0.14 |

^1^n = 10 pigs per treatment except for SF- with 8 pigs.

**Table S10.** Relative abundance of bacterial genus in **colonic digesta** of pigs fed control diet without ETEC (NC), control diet with ETEC (PC), soluble fiber diet without (SF-) or with enzymes (SF+), or insoluble fiber diet without (IF-) or with enzymes (IF+) after an ETEC F18 challenge, %^1^

| Taxonomy |  | Treatment | | | | | | | |
| --- | --- | --- | --- | --- | --- | --- | --- | --- | --- |
|  |  | NC | PC |  | SF | |  | IF | |
|  |  |  |  |  | SF- | SF+ |  | IF- | IF+ |
| ***Bacteroidetes*** |  |  |  |  |  |  |  |  |  |
| *Bacteroidaceae* | *Bacteroides* | 0.27 | 1.39 |  | 1.53 | 0.29 |  | 0.15 | 0.48 |
| *Bacteroidales S24-7 group* | *Bacteroidales S24-7 group ge* | 2.27 | 2.68 |  | 1.31 | 2.28 |  | 2.81 | 3.05 |
| *Porphyromonadaceae* | *Odoribacter* | 0.00 | 1.07 |  | 0.20 | 0.00 |  | 0.01 | 0.01 |
| *Porphyromonadaceae* | *Parabacteroides* | 0.16 | 0.49 |  | 1.02 | 0.23 |  | 0.45 | 0.48 |
| *Prevotellaceae* | *Alloprevotella* | 2.52 | 7.87 |  | 6.02 | 3.04 |  | 5.16 | 9.22 |
| *Prevotellaceae* | *Prevotella 7* | 1.16 | 0.03 |  | 2.15 | 0.63 |  | 0.38 | 0.04 |
| *Prevotellaceae* | *Prevotella 9* | 2.92 | 1.14 |  | 2.50 | 3.15 |  | 4.73 | 2.58 |
| *Prevotellaceae* | *Prevotellaceae NK3B31 group* | 0.82 | 1.12 |  | 1.37 | 2.43 |  | 1.15 | 1.99 |
| *Prevotellaceae* | *Prevotellaceae UCG 001* | 0.10 | 0.06 |  | 0.41 | 0.04 |  | 0.25 | 1.02 |
| *Prevotellaceae* | *Prevotellaceae UCG 003* | 1.11 | 2.72 |  | 1.64 | 0.62 |  | 2.25 | 2.05 |
| *Prevotellaceae* | *uncultured* | 0.11 | 0.15 |  | 0.09 | 0.07 |  | 0.89 | 1.49 |
| *Rikenellaceae* | *Rikenellaceae RC9 gut group* | 3.37 | 2.70 |  | 3.73 | 7.43 |  | 4.40 | 3.81 |
| ***Firmicutes*** |  |  |  |  |  |  |  |  |  |
| *Acidaminococcaceae* | *Phascolarctobacterium* | 17.26 | 14.55 |  | 16.31 | 12.13 |  | 19.09 | 19.85 |
| *Christensenellaceae* | *Christensenellaceae R-7 group* | 0.51 | 0.45 |  | 0.41 | 1.00 |  | 0.42 | 0.43 |
| *Lachnospiraceae* | *Blautia* | 1.01 | 0.72 |  | 1.51 | 2.48 |  | 1.25 | 0.89 |
| *Lachnospiraceae* | *Coprococcus 1* | 0.93 | 0.28 |  | 0.09 | 0.39 |  | 0.55 | 1.04 |
| *Lachnospiraceae* | *Coprococcus 3* | 1.14 | 0.77 |  | 0.69 | 1.68 |  | 0.70 | 0.81 |
| *Lachnospiraceae* | *Lachnoclostridium* | 0.06 | 2.31 |  | 0.25 | 0.03 |  | 0.04 | 0.08 |
| *Lachnospiraceae* | *Roseburia* | 1.03 | 1.28 |  | 1.86 | 1.95 |  | 3.14 | 1.15 |
| *Lactobacillaceae* | *Lactobacillus* | 18.35 | 5.41 |  | 5.62 | 11.77 |  | 10.50 | 5.66 |
| *Ruminococcaceae* | *Faecalibacterium* | 2.64 | 0.67 |  | 0.78 | 2.18 |  | 1.22 | 1.18 |
| *Ruminococcaceae* | *Ruminococcaceae NK4A214 group* | 0.97 | 0.64 |  | 1.05 | 1.70 |  | 0.93 | 1.29 |
| *Ruminococcaceae* | *Ruminococcaceae UCG 002* | 2.04 | 1.47 |  | 2.08 | 3.14 |  | 1.55 | 2.15 |
| *Ruminococcaceae* | *Ruminococcaceae UCG 005* | 0.96 | 0.61 |  | 0.86 | 1.40 |  | 1.26 | 1.26 |
| *Ruminococcaceae* | *Ruminococcaceae UCG 014* | 0.52 | 0.28 |  | 0.56 | 1.45 |  | 0.44 | 0.58 |
| *Ruminococcaceae* | *Ruminococcus 1* | 1.43 | 0.79 |  | 1.77 | 0.99 |  | 1.34 | 1.71 |
| *Ruminococcaceae* | *Subdoligranulum* | 1.17 | 1.02 |  | 0.50 | 1.82 |  | 0.61 | 0.61 |
| *Streptococcaceae* | *Streptococcus* | 0.03 | 0.10 |  | 0.13 | 1.06 |  | 0.09 | 0.05 |
| *Veillonellaceae* | *Anaerovibrio* | 3.62 | 0.80 |  | 3.41 | 0.78 |  | 2.28 | 1.32 |
| *Veillonellaceae* | *Megasphaera* | 12.73 | 10.55 |  | 4.99 | 2.78 |  | 8.06 | 2.39 |
| *Veillonellaceae* | *Schwartzia* | 0.00 | 0.00 |  | 0.00 | 2.09 |  | 0.00 | 0.00 |
| *Veillonellaceae* | *Selenomonas* | 0.13 | 0.04 |  | 0.02 | 0.12 |  | 1.06 | 0.08 |
| *Veillonellaceae* | *Veillonella* | 0.18 | 0.40 |  | 1.71 | 0.02 |  | 0.41 | 0.96 |
| ***Proteobacteria*** |  |  |  |  |  |  |  |  |  |
| *Enterobacteriaceae* | *Escherichia-Shigella* | 0.03 | 4.10 |  | 6.82 | 0.94 |  | 0.71 | 2.33 |
| ***Spirochaetae*** |  |  |  |  |  |  |  |  |  |
| *Spirochaetaceae* | *Treponema 2* | 0.46 | 1.71 |  | 1.13 | 1.39 |  | 1.71 | 3.10 |
| ***Verrucomicrobia*** |  |  |  |  |  |  |  |  |  |
| *Verrucomicrobiaceae* | *Akkermansia* | 0.01 | 6.96 |  | 0.00 | 0.05 |  | 0.01 | 0.14 |

^1^n = 10 pigs per treatment except for SF- with 8 pigs.

**Table S11.** Differential abundance in specific taxa in **ileal digesta** of pigs fed control diet without ETEC (NC), control diet with ETEC (PC), soluble fiber diet without (SF-) or with enzymes (SF+), or insoluble fiber diet without (IF-) or with enzymes (IF+) after an ETEC F18 challenge according to DESeq2

| Treatments compared | BaseMean^1^ | Log2(fold change) | lfcSE^2^ | padj^3^ | Taxonomic annotation |
| --- | --- | --- | --- | --- | --- |
| NC over PC | 35409.51 | -9.30 | 1.17 | 0.0000000 | *Enterobacteriaceae* |
|  | 20021.91 | -7.91 | 1.17 | 0.0000000 | *Escherichia-Shigella* |
| SF- over PC | 969.53 | -6.34 | 1.48 | 0.0002632 | *Pasteurellaceae* |
|  | 262.75 | 3.28 | 0.80 | 0.0003861 | *Erysipelotrichaceae* |
|  | 1471.90 | 3.14 | 0.95 | 0.0037949 | *Streptococcaceae* |
|  | 2112.30 | 1.82 | 0.76 | 0.0528013 | *Prevotellaceae* |
|  | 311.81 | 5.05 | 1.20 | 0.0003874 | *Roseburia* |
|  | 1537.63 | 4.22 | 1.01 | 0.0004177 | *Streptococcus* |
|  | 169.11 | 4.97 | 1.52 | 0.0068686 | *Turicibacter* |
|  | 1063.19 | 3.95 | 1.22 | 0.0078043 | *Clostridium_sensu_stricto_1* |
|  | 654.58 | -4.97 | 1.59 | 0.0099225 | *Actinobacillus* |
| SF+ over PC | 262.75 | 4.91 | 0.79 | 0.0000000 | *Erysipelotrichaceae* |
|  | 1471.90 | 5.54 | 0.95 | 0.0000001 | *Streptococcaceae* |
|  | 1567.39 | 4.43 | 1.12 | 0.0009044 | *Clostridiaceae 1* |
|  | 2112.30 | 2.18 | 0.76 | 0.0288947 | *Prevotellaceae* |
|  | 35409.51 | -2.88 | 1.17 | 0.0747572 | *Enterobacteriaceae* |
|  | 1537.63 | 6.17 | 1.01 | 0.0000000 | *Streptococcus* |
|  | 1063.19 | 6.74 | 1.22 | 0.0000004 | *Clostridium_sensu_stricto_1* |
|  | 169.11 | 5.29 | 1.52 | 0.0032939 | *Turicibacter* |
|  | 311.81 | 3.49 | 1.23 | 0.0215195 | *Roseburia* |
|  | 20021.91 | -2.60 | 1.17 | 0.0996602 | *Escherichia-Shigella* |
| IF- over PC | 262.75 | -2.98 | 0.89 | 0.0097459 | *Erysipelotrichaceae* |
|  | 35409.51 | -2.99 | 1.20 | 0.0696702 | *Enterobacteriaceae* |
|  | 169.11 | -4.22 | 1.65 | 0.0624969 | *Turicibacter* |
| IF+ over PC | 1269.51 | 5.55 | 0.73 | 0.0000000 | *Lachnospiraceae* |
|  | 1567.39 | 4.39 | 1.12 | 0.0012876 | *Clostridiaceae 1* |
|  | 2112.30 | 1.99 | 0.76 | 0.0635553 | *Prevotellaceae* |
|  | 311.81 | 12.88 | 1.16 | 0.0000000 | *Roseburia* |
|  | 1063.19 | 6.42 | 1.22 | 0.0000018 | *Clostridium sensu stricto 1* |
|  | 32760.24 | 1.84 | 0.73 | 0.0431480 | *Lactobacillus* |
| SF+ over SF- | 35409.51 | -3.58 | 1.17 | 0.0346408 | *Enterobacteriaceae* |
|  | 1567.39 | 3.41 | 1.12 | 0.0346408 | *Clostridiaceae 1* |
|  | 969.53 | 4.15 | 1.48 | 0.0609233 | *Pasteurellaceae* |
|  | 20021.91 | -4.89 | 1.17 | 0.0008861 | *Escherichia-Shigella* |
| IF+ over IF- | 1269.51 | 6.18 | 0.75 | 0.0000000 | *Lachnospiraceae* |
|  | 262.75 | 4.72 | 0.88 | 0.0000011 | *Erysipelotrichaceae* |
|  | 2112.30 | 3.66 | 0.79 | 0.0000375 | *Prevotellaceae* |
|  | 1567.39 | 4.38 | 1.15 | 0.0008856 | *Clostridiaceae_1* |
|  | 311.81 | 10.57 | 1.14 | 0.0000000 | *Roseburia* |
|  | 1063.19 | 9.09 | 1.29 | 0.0000000 | *Clostridium sensu stricto 1* |
|  | 169.11 | 6.91 | 1.64 | 0.0002065 | *Turicibacter* |

^1^Mean of normalized counts for all samples.

^2^Standard error of DESeq2.

^3^padj is the Benjamini–Hochberg adjusted *P* value.

**Table S12.** Differential abundance in specific taxa in **colonic digesta** of pigs fed control diet without ETEC (NC), control diet with ETEC (PC), soluble fiber diet without (SF-) or with enzymes (SF+), or insoluble fiber diet without (IF-) or with enzymes (IF+) after an ETEC F18 challenge according to DESeq2

| Treatments compared | BaseMean^1^ | Log2(fold change) | lfcSE^2^ | padj^3^ | Taxonomic annotation |
| --- | --- | --- | --- | --- | --- |
| NC over PC | 35409.51 | -6.12 | 1.18 | 0.0000039 | *Enterobacteriaceae* |
|  | 83.88 | -3.30 | 0.93 | 0.0059766 | *Porphyromonadaceae* |
|  | 49574.35 | 2.45 | 0.76 | 0.0147178 | *Lactobacillaceae* |
|  | 20021.91 | -6.64 | 1.17 | 0.0000004 | *Escherichia-Shigella* |
|  | 60.57 | -5.59 | 1.07 | 0.0000034 | *Lachnoclostridium* |
|  | 32760.24 | 2.23 | 0.73 | 0.0279047 | *Lactobacillus* |
|  | 134.80 | 5.44 | 1.97 | 0.0592766 | *Prevotella_7* |
|  | 284.49 | 2.94 | 1.12 | 0.0794783 | *Anaerovibrio* |
| SF- over PC | 1.93 | -24.81 | 3.71 | 0.0000000 | *Odoribacter* |
|  | 134.80 | 7.71 | 2.09 | 0.0210033 | *Prevotella_7* |
|  | 60.57 | -3.77 | 1.13 | 0.0380848 | *Lachnoclostridium* |
| SF+ over PC | 83.88 | -2.96 | 0.93 | 0.0300813 | *Porphyromonadaceae* |
|  | 1471.90 | 2.78 | 0.95 | 0.0486241 | *Streptococcaceae* |
|  | 1.93 | -23.62 | 3.49 | 0.0000000 | *Odoribacter* |
|  | 60.57 | -6.78 | 1.07 | 0.0000000 | *Lachnoclostridium* |
|  | 440.15 | -4.63 | 1.25 | 0.0058850 | *Veillonella* |
|  | 1537.63 | 3.00 | 1.02 | 0.0555725 | *Streptococcus* |
|  | 206.61 | 1.27 | 0.46 | 0.0579990 | *Ruminococcaceae_NK4A214_group* |
|  | 96.93 | 2.22 | 0.82 | 0.0614201 | *Ruminococcaceae_UCG 014* |
|  | 213.15 | -2.24 | 0.87 | 0.0831071 | *Prevotellaceae UCG 003* |
| IF- over PC | 78.17 | -3.06 | 1.09 | 0.0994062 | *Bacteroidaceae* |
|  | 60.57 | -6.29 | 1.07 | 0.0000004 | *Lachnoclostridium* |
|  | 117.68 | -3.61 | 1.13 | 0.0537367 | *Bacteroides* |
| IF+ over PC | 60.57 | -5.28 | 1.07 | 0.0001313 | *Lachnoclostridium* |
| SF+ over SF- | 440.15 | -6.26 | 1.32 | 0.0003848 | *Veillonella* |

^1^Mean of normalized counts for all samples.

^2^Standard error of DESeq2.

^3^padj is the Benjamini–Hochberg adjusted *P* value.

**Table S13.** Correlation coefficients between volatile fatty acids and most abundant microbial taxa (> 1% in at least one treatment) in the **colonic digesta**^1^

| Bacteria | Volatile fatty acids | R | *P*-value |
| --- | --- | --- | --- |
| *Lachnoclostridium* | Acetic acid | -0.35 | 0.074 |
|  | Propionic acid | -0.41 | 0.031 |
| *Blautia* | Acetic acid | 0.41 | 0.031 |
| *Ruminococcaceae UCG 014* | Butyric acid | 0.37 | 0.065 |
|  | Isobutyric acid | -0.36 | 0.068 |
| *Treponema 2* | Valeric acid | -0.36 | 0.067 |
| *Akkermansia* | Propionic acid | -0.36 | 0.068 |
| *Prevotella 9* | Butyric acid | 0.48 | <0.001 |
|  | Valeric acid | 0.44 | 0.018 |
| *Prevotella 7* | Butyric acid | 0.44 | 0.017 |
|  | Valeric acid | 0.39 | 0.044 |
| *Bacteroides* | Acetic acid | -0.36 | 0.067 |
|  | Propionic acid | -0.47 | 0.009 |
|  | Butyric acid | -0.50 | 0.006 |
|  | Valeric acid | -0.46 | 0.013 |
| *Selenomonas* | Valeric acid | 0.40 | 0.039 |
| *Megasphaera* | Valeric acid | 0.59 | <0.001 |

^1^Only significant and tendency for significant correlations were shown.

**Table S14.** Growth performance and diarrhea incidence in pigs fed control diet without ETEC (NC), control diet with ETEC (PC), soluble fiber diet without (SF-) or with enzymes (SF+), or insoluble fiber diet without (IF-) or with enzymes (IF+) after an ETEC F18 challenge, %

| Item | Treatment | | | | | | | | SEM | Contrast *P*-value^1^ | | |
| --- | --- | --- | --- | --- | --- | --- | --- | --- | --- | --- | --- | --- |
|  | NC | PC |  | SF | |  | IF | |  |  |  |  |
|  |  |  |  | SF- | SF+ |  | IF- | IF+ |  | Fiber | Enzyme | F × E |
| Pig no. | 10 | 8 |  | 10 | 10 |  | 10 | 10 |  |  |  |  |
| BW, kg |  |  |  |  |  |  |  |  |  |  |  |  |
| dpi -7 | 6.86 | 6.84 |  | 7.03 | 6.89 |  | 6.93 | 6.84 | 0.25 | 0.416 | 0.220 | 0.812 |
| dpi 0 | 7.87 | 7.36 |  | 8.07 | 8.40^*^ |  | 8.22^#^ | 7.52 | 0.40 | 0.201 | 0.520 | 0.078 |
| dpi 7 | 9.90^*^ | 8.57 |  | 9.82^#^ | 10.34^*^ |  | 9.93^*^ | 9.17 | 0.58 | 0.211 | 0.777 | 0.136 |
| dpi -7 to 0 |  |  |  |  |  |  |  |  |  |  |  |  |
| ADG, kg | 0.14 | 0.09 |  | 0.15 | 0.22^*^ |  | 0.18 | 0.10 | 0.04 | 0.305 | 0.833 | 0.063 |
| ADFI, kg | 0.24 | 0.17 |  | 0.18 | 0.24 |  | 0.21 | 0.16 | 0.03 | 0.404 | 0.974 | 0.076 |
| G:F | 0.78 | 0.60 |  | 0.87 | 0.91^#^ |  | 0.86 | 0.66 | 0.01 | 0.210 | 0.435 | 0.245 |
| dpi 1 to 7 |  |  |  |  |  |  |  |  |  |  |  |  |
| ADG, kg | 0.29^*^ | 0.17 |  | 0.25^#^ | 0.28^*^ |  | 0.244^#^ | 0.236 | 0.03 | 0.397 | 0.731 | 0.527 |
| ADFI, kg | 0.45^*^ | 0.31 |  | 0.39 | 0.43^#^ |  | 0.37 | 0.36 | 0.05 | 0.294 | 0.806 | 0.590 |
| G:F | 0.69 | 0.55 |  | 0.53 | 0.64 |  | 0.62 | 0.69 | 0.08 | 0.341 | 0.196 | 0.775 |
| Overall |  |  |  |  |  |  |  |  |  |  |  |  |
| ADG, kg | 0.22^*^ | 0.13 |  | 0.20 | 0.25^*^ |  | 0.22^#^ | 0.17 | 0.03 | 0.266 | 0.986 | 0.107 |
| ADFI, kg | 0.31^#^ | 0.23 |  | 0.27 | 0.32^#^ |  | 0.28 | 0.24 | 0.03 | 0.294 | 0.843 | 0.208 |
| G:F | 0.62 | 0.51 |  | 0.76^*^ | 0.76^*^ |  | 0.65 | 0.65 | 0.18 | 0.179 | 0.981 | 0.950 |
| Diarrhea, %^2^ |  |  |  |  |  |  |  |  |  |  |  |  |
| dpi -7 to 0 | 0 | 0 |  | 5.71 | 2.86 |  | 5.71 | 1.43 | - | 0.622 | 0.133 | 0.622 |
| dpi 1 to 7 | 7.14* | 40.00 |  | 31.43 | 27.14 |  | 57.14* | 40.00 | - | <0.001 | 0.075 | 0.337 |

^*^Indicates significant difference compared with PC (*P* ≤ 0.05).

^#^Indicates tendency for difference compared with PC (0.05 < *P* ≤ 0.10).

^1^Fiber: dietary fiber type effect (SF vs. IF); Enzyme: carbohydrases effect (without vs. with); F × E: fiber type by carbohydrases interaction effect.

^2^Diarrhea incidence (%) = (total number of pigs with diarrhea score ≥ 3) / (total number of pigs) × 100; statistical analysis was conducted by a chi-squared test.

**Supplementary Figures**

**Fig. S1.** LEfSe reveals predicted biological effect sizes of differential taxa in **fecal microbiota** of pigs fed basal control diets, soluble fiber diets without (SF-) or with enzymes (SF+), or insoluble fiber diets without (IF-) or with enzymes (IF+). Histogram of the discriminant analysis (LDA) scores computed for features differentially abundant bacteria between (**A**) Basal and SF-; (**B)** Basal and SF+; (**C)** Basal and IF-; (**D**) Basal and IF+. Differences are represented by the color of the treatment in which specific taxa were most abundant.

**Fig. S2.** LEfSe reveals predicted biological effect sizes of differential taxa in **colonic microbiota** of pigs fed control diet without ETEC (NC), control diet with ETEC (PC), soluble fiber diet without (SF-) or with enzymes (SF+), or insoluble fiber diet without (IF-) or with enzymes (IF+) after an F18 ETEC challenge. Histogram of the discriminant analysis (LDA) scores computed for features differentially abundant bacteria and Cladogram revealing statistically and biologically consistent differences in detected taxa between (**A, B**) NC and PC, (**C, D**) PC and SF-, (**E, F**) PC and SF+, (**G, H**) PC and IF-, and (**I, J**) PC and IF+. Differences are represented by the color of the treatment in which specific taxa were most abundant and the diameter of each circle is proportional to the relative abundance.

**Fig. S3.** Predicted functional metagenomes of **colonic digesta microbiota** of pigs fed control diet without ETEC (NC), control diet with ETEC (PC), soluble fiber diet without (SF-) or with enzymes (SF+), or insoluble fiber diet without (IF-) or with enzymes (IF+) after an F18 ETEC challenge. (**A**) NC and PC; (**B**) PC and SF-; (**C**) PC and SF+; (**D**) PC and IF-; (**E**) PC and IF+. Linear discriminant analysis (LDA) was performed to identify significant changes in the proportion of reconstructed functional pathways obtained from PICRUSt predictive algorithms at Kyoto Encyclopaedia of Genes and Genomes (KEGG). Analysis was performed using linear discriminant analysis of effect size (LEfSe), a metagenome analysis approach which performs the LDA following the Wilcoxon Mann-Whitney test to assess effect size of each differentially abundant variable.

B. Basal vs. SF+

**Fig. S1**


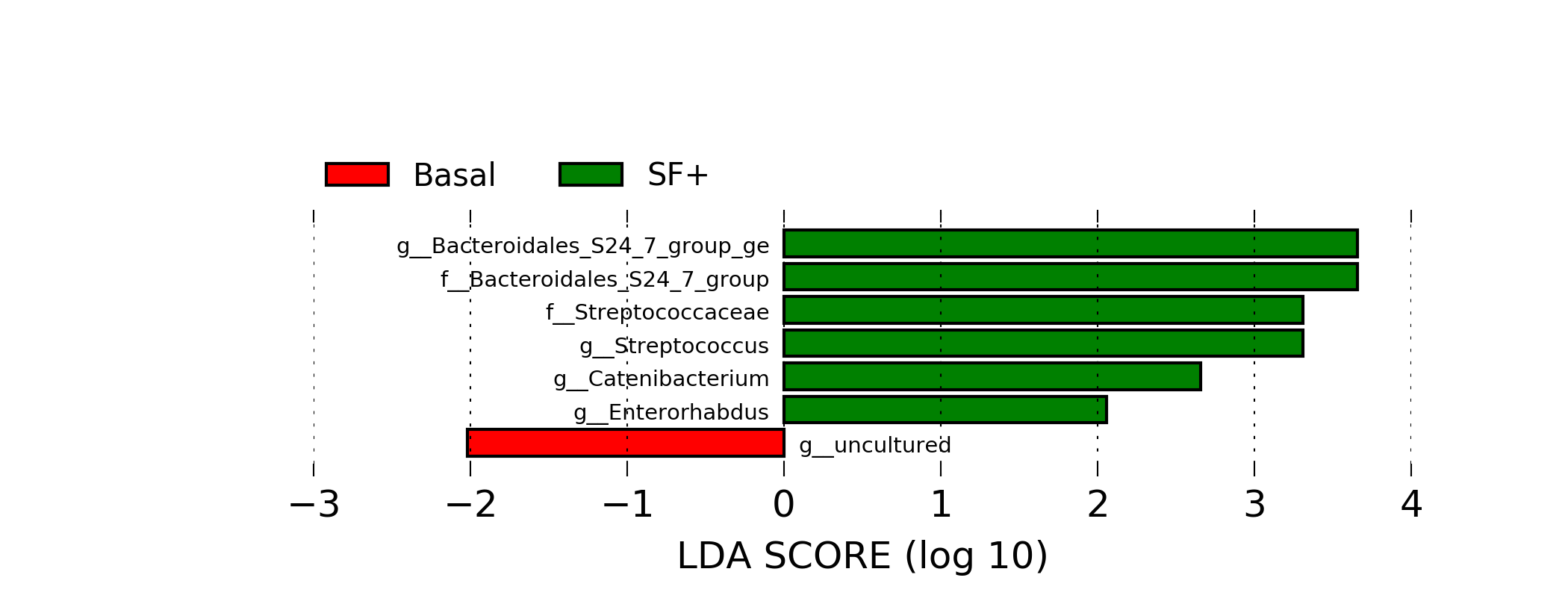

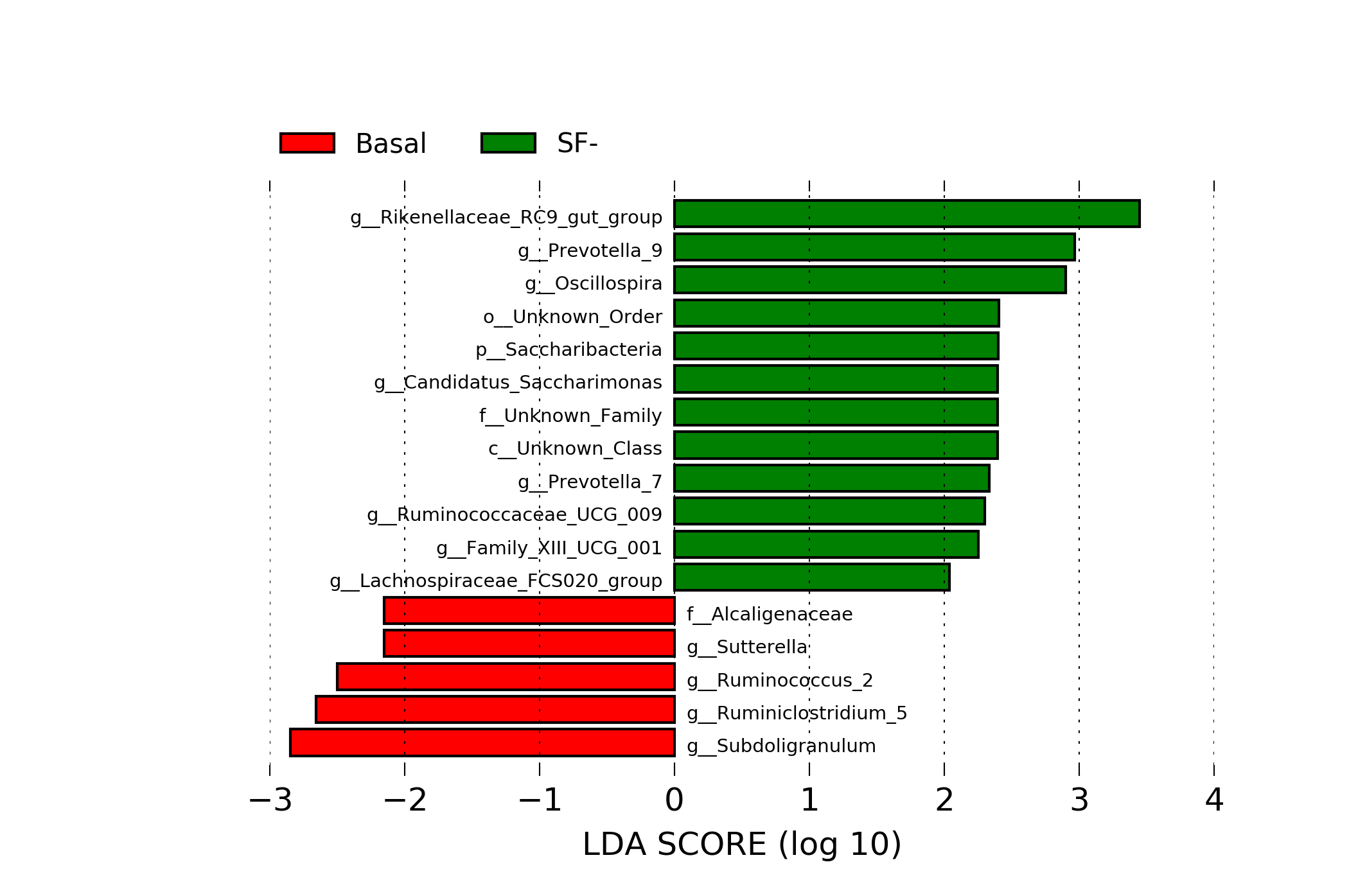


D. Basal vs. IF+

C. Basal vs. IF-

A. Basal vs. SF-


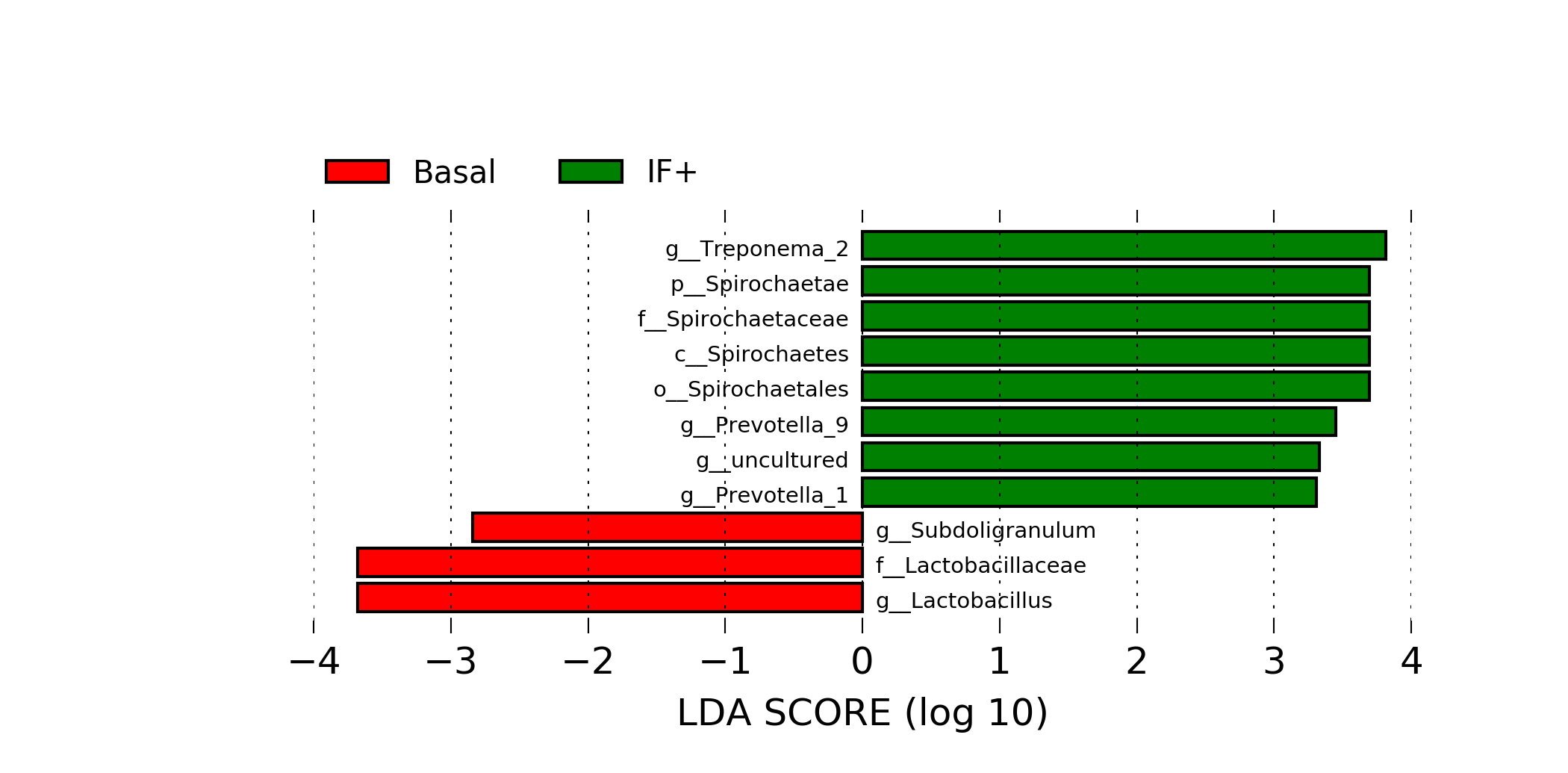

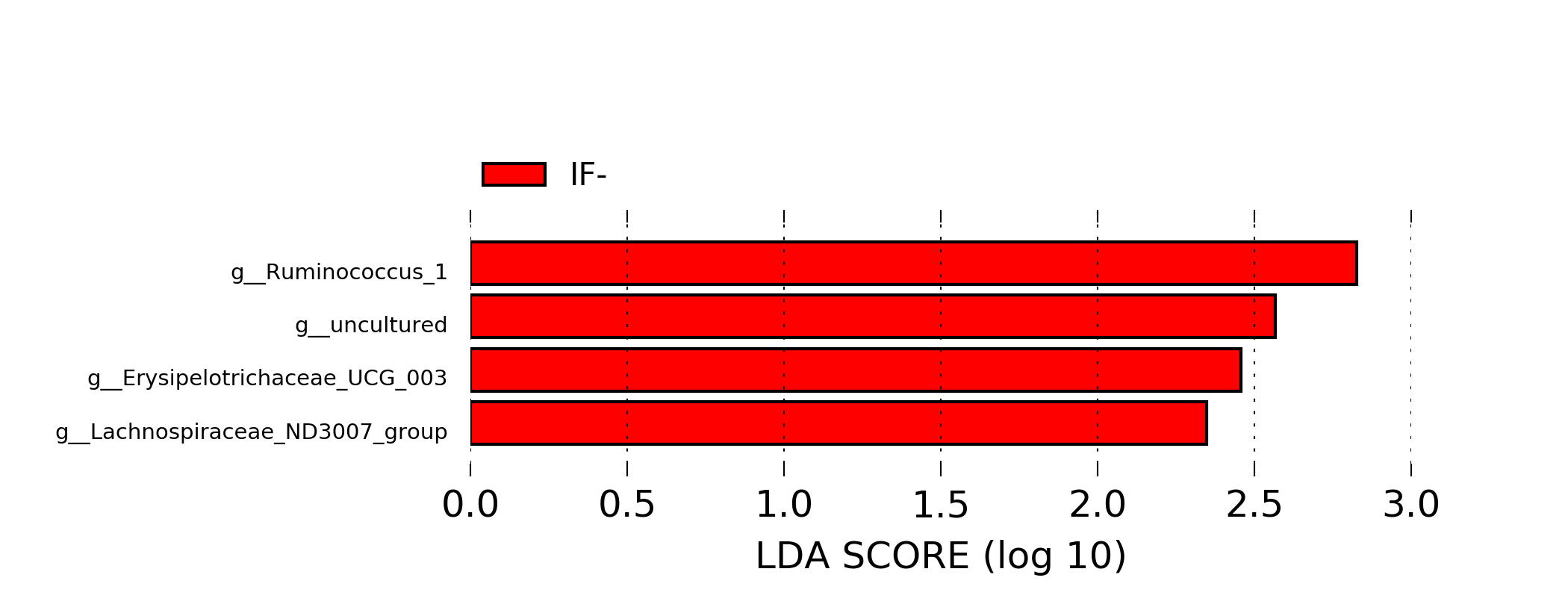


**Fig. S2**


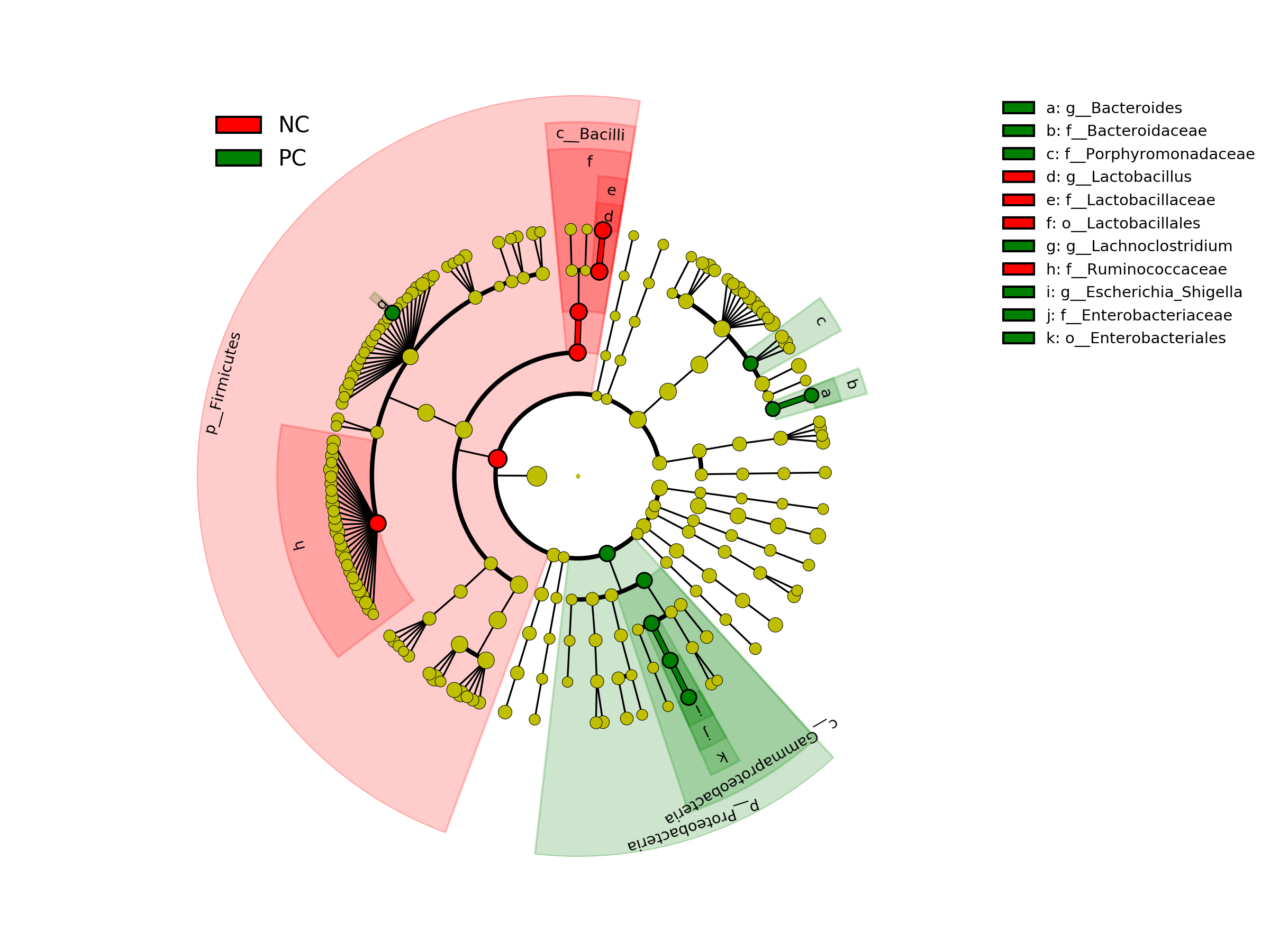


B. NC vs. PC

A. NC vs. PC


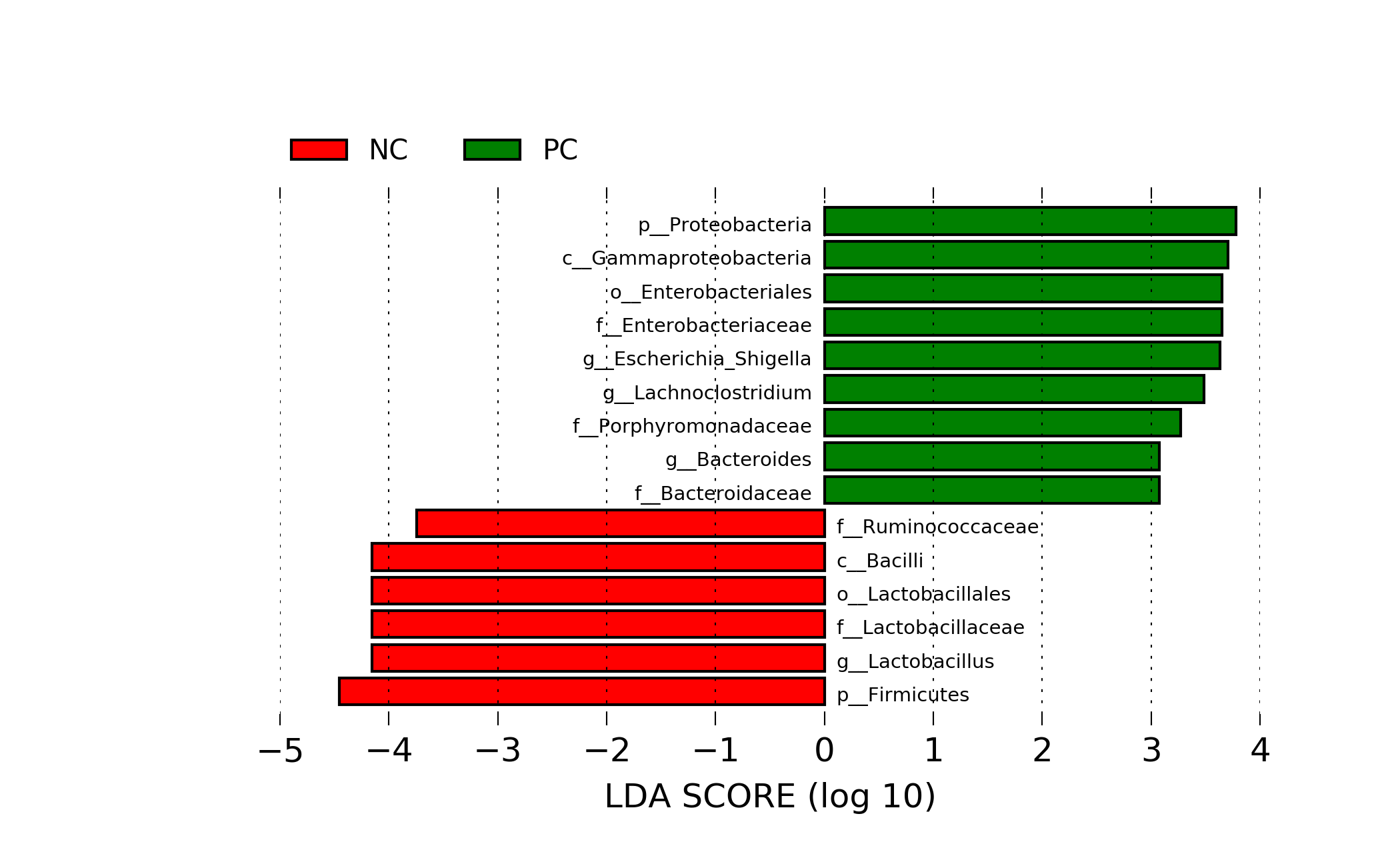


C. PC vs. SF-

D. PC vs. SF-


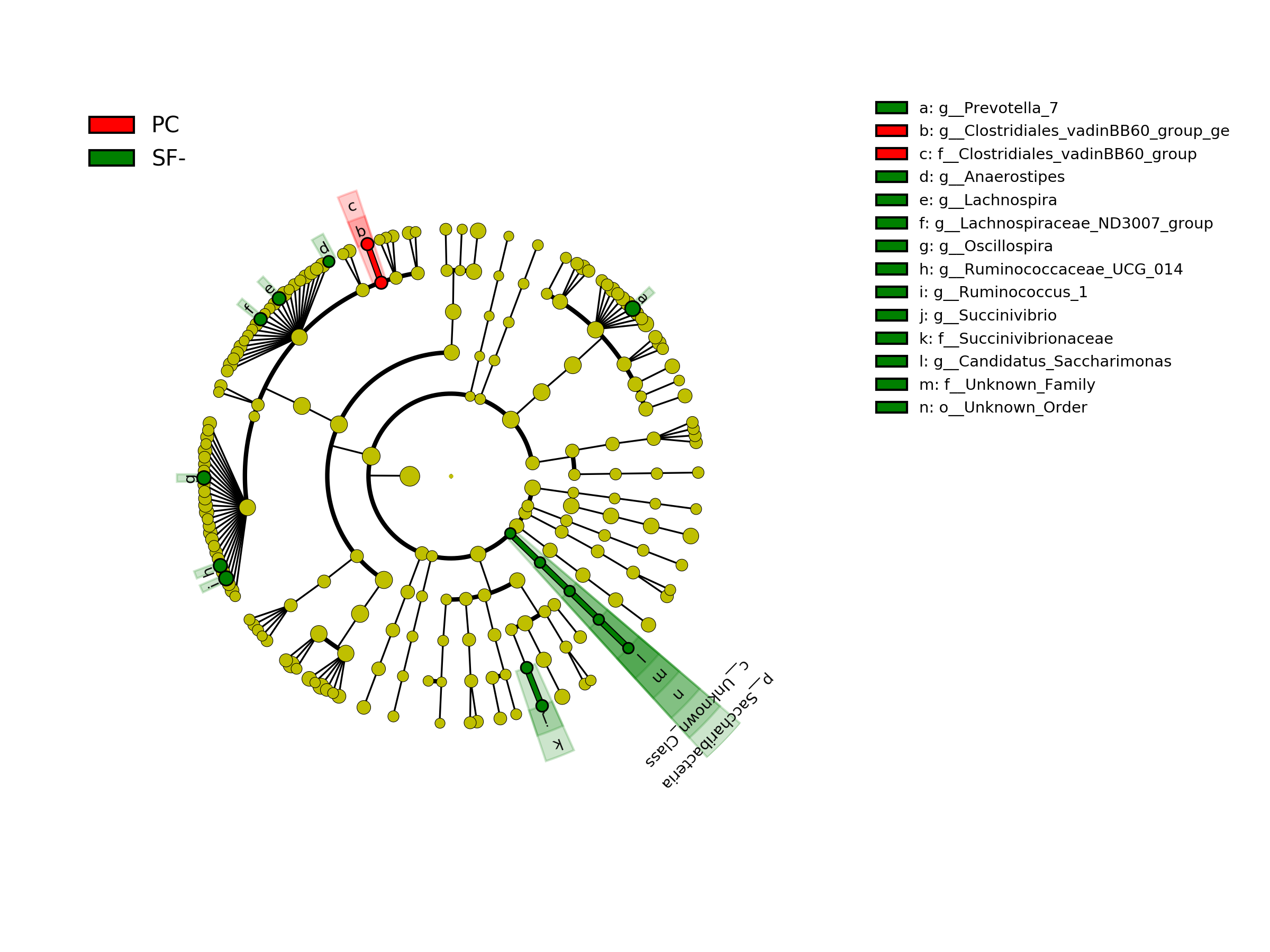

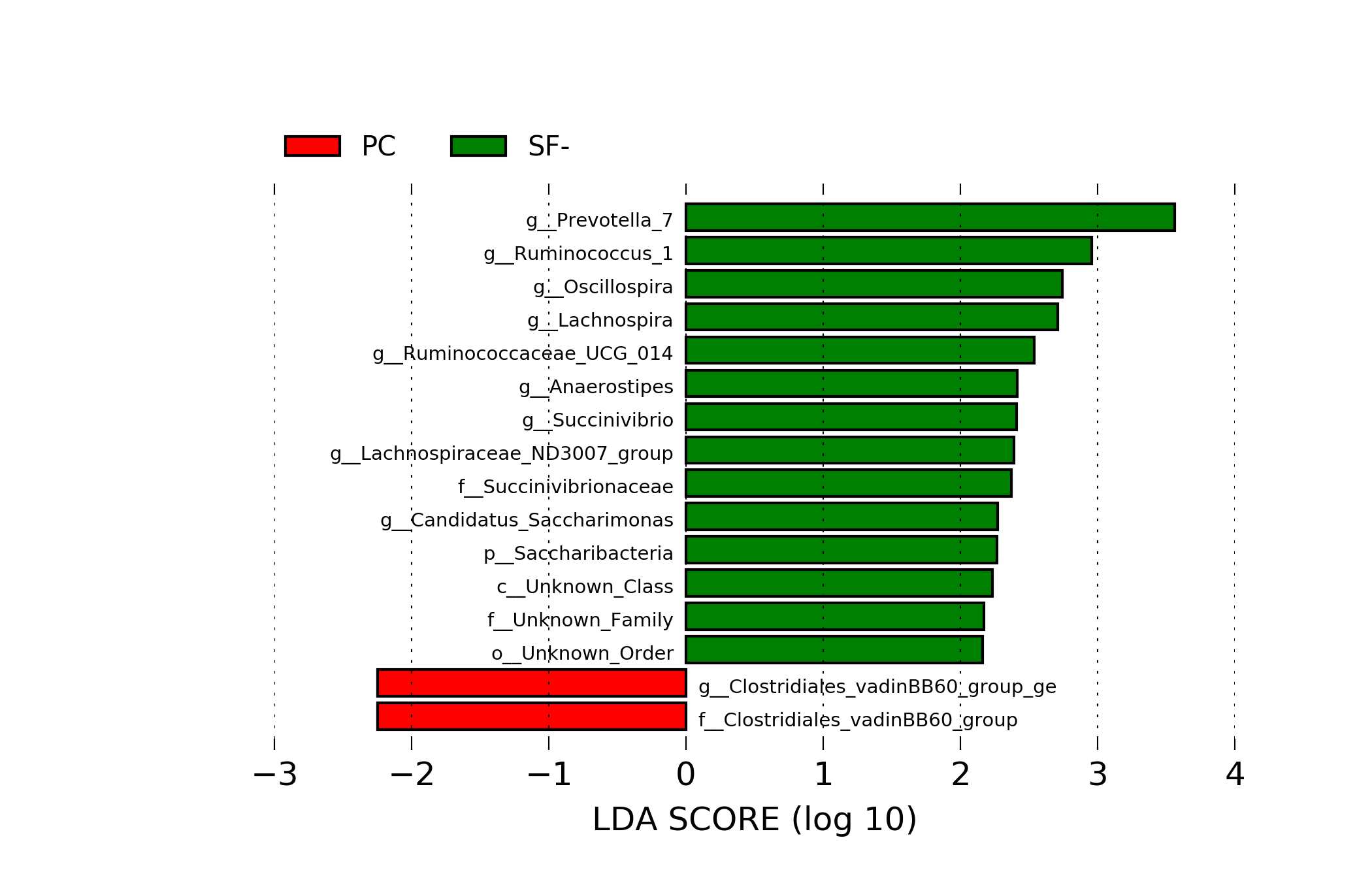


F. PC vs. SF+

E. PC vs. SF+


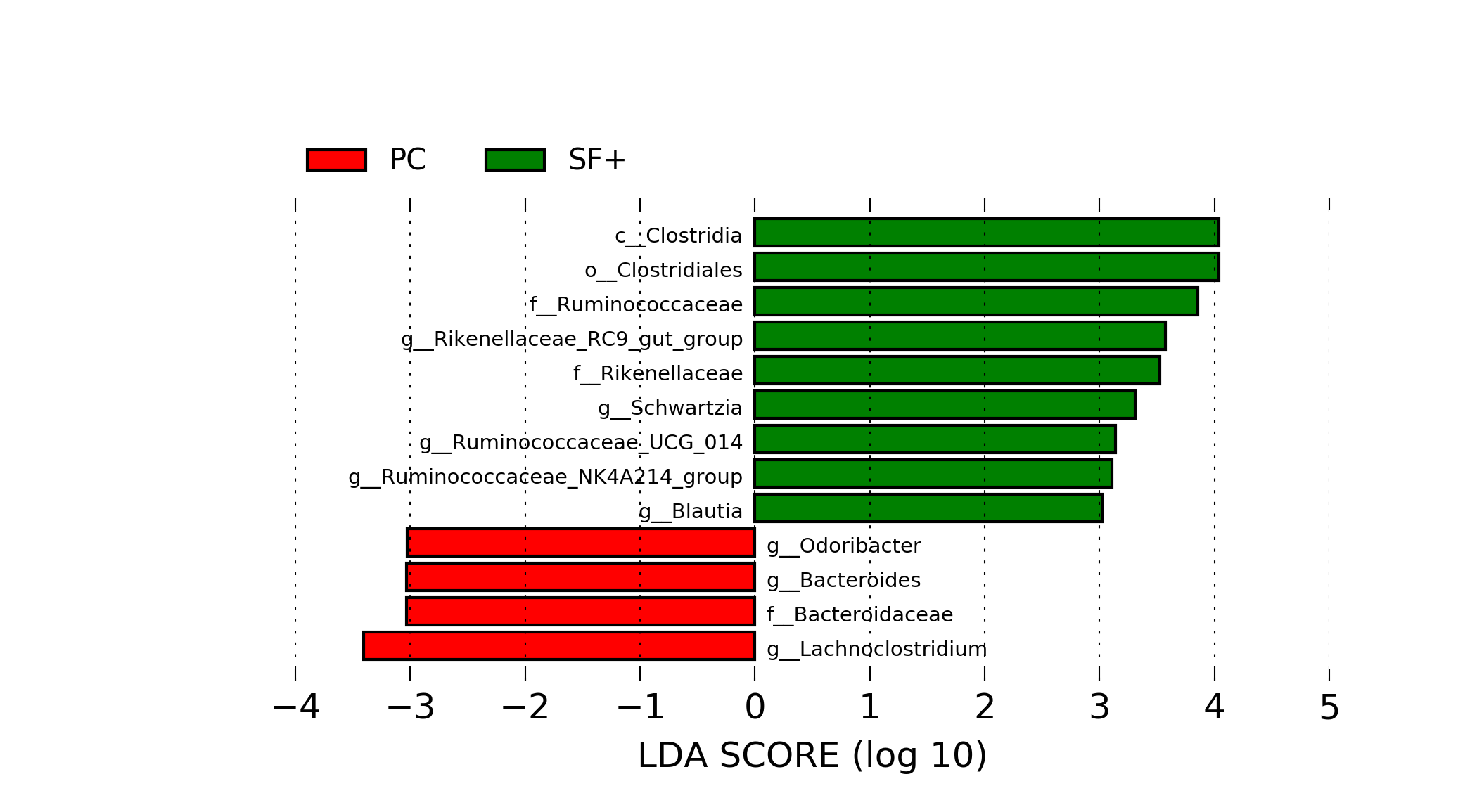

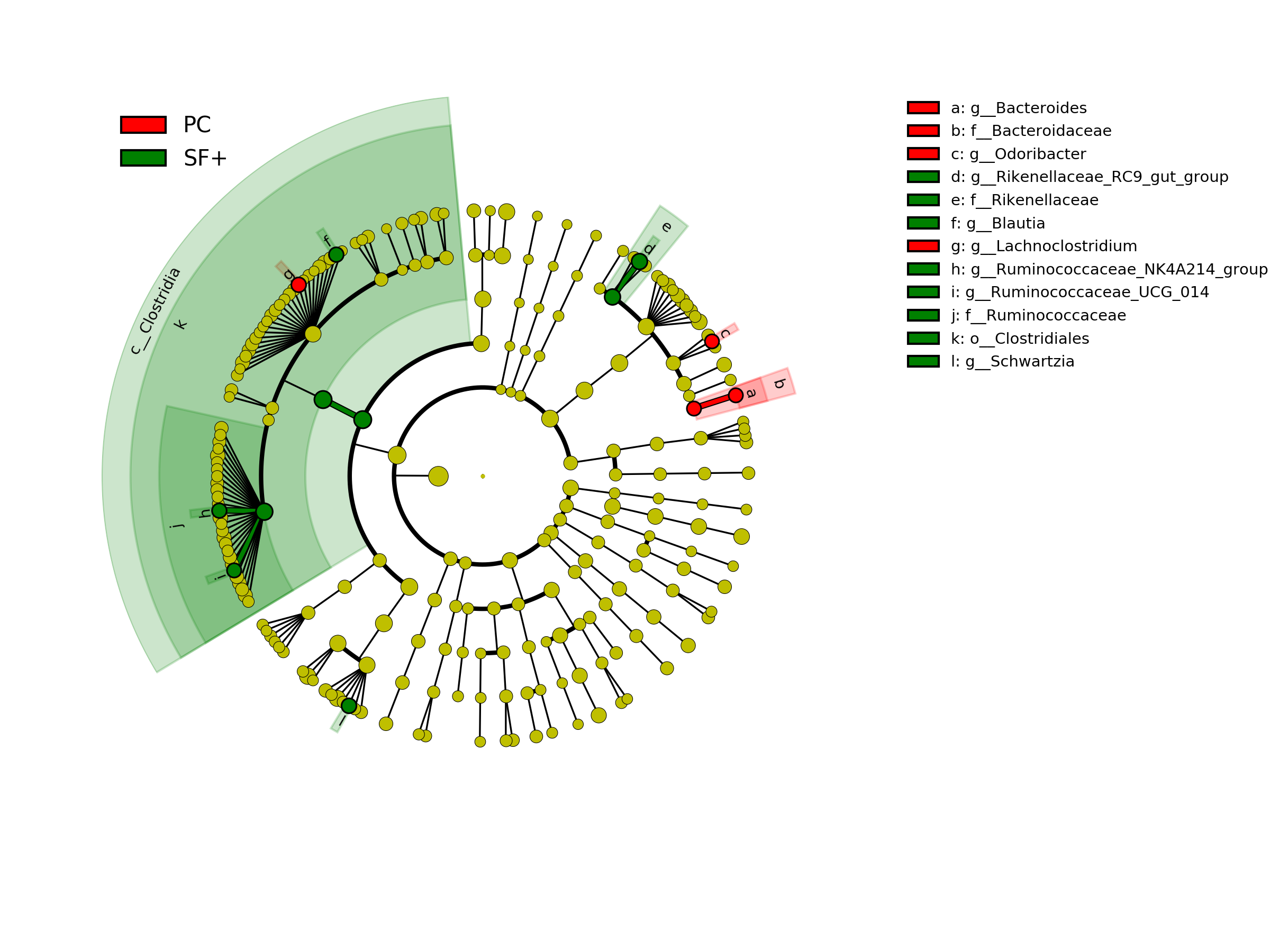


H. PC vs. IF-

G. PC vs. IF-


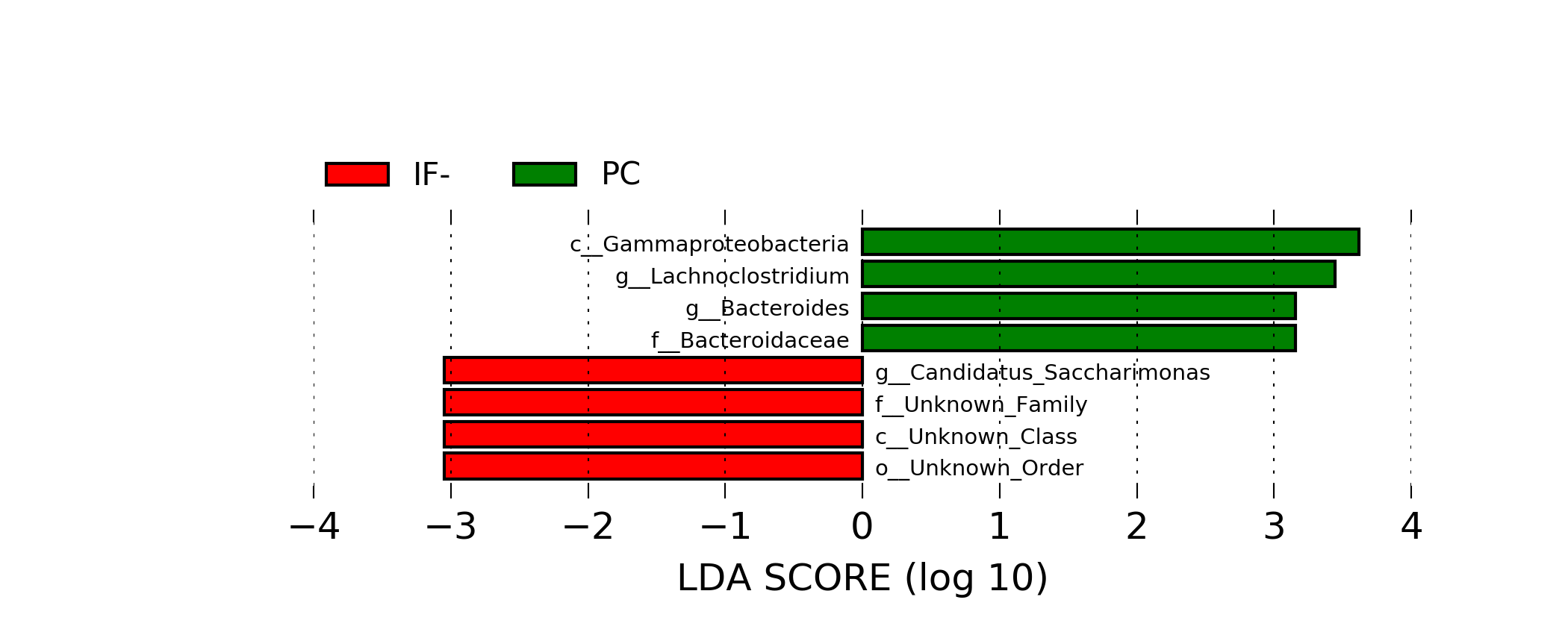

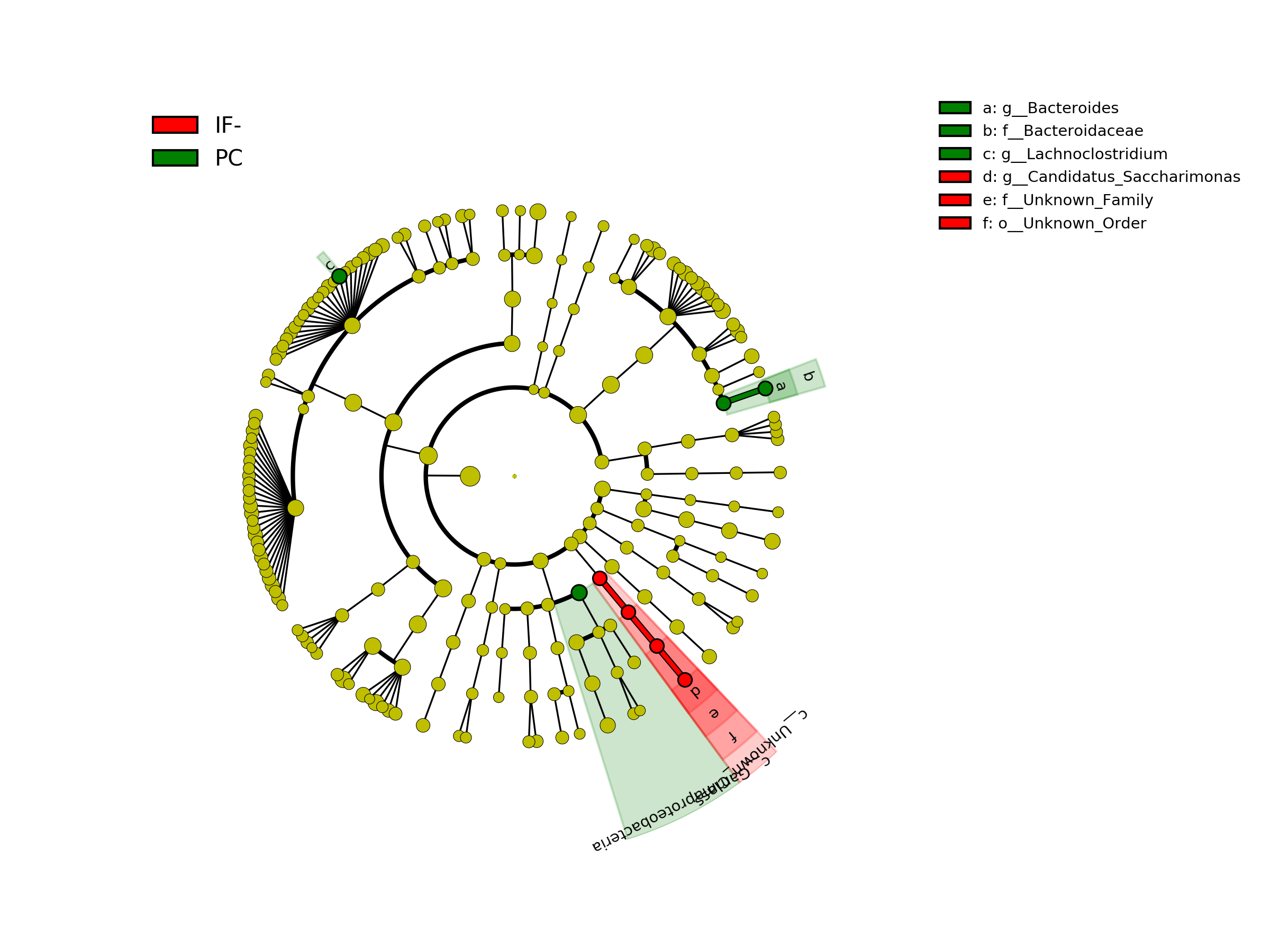


J. PC vs. IF+

I. PC vs. IF+


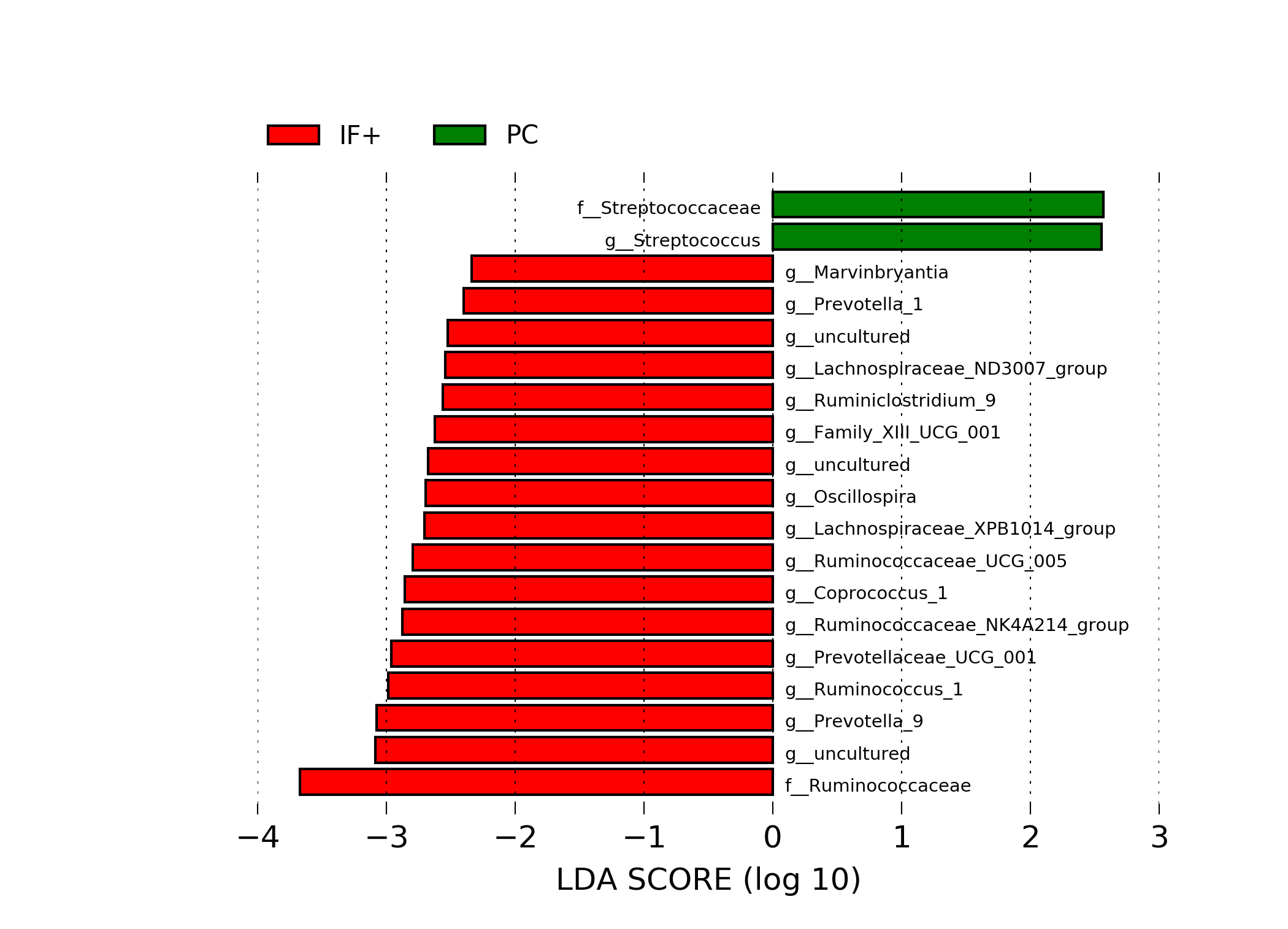

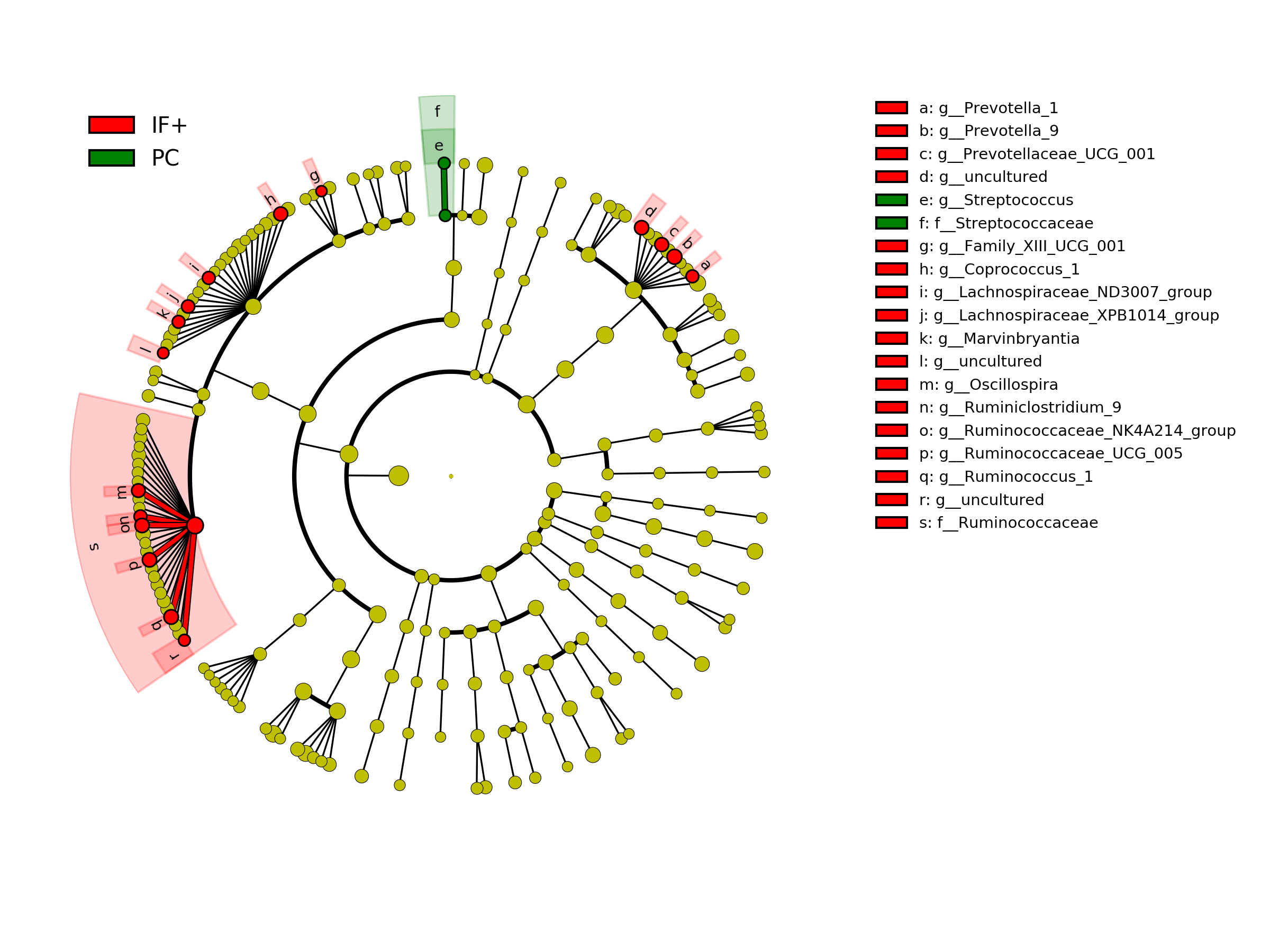


**Fig. S3**

A. NC vs. PC


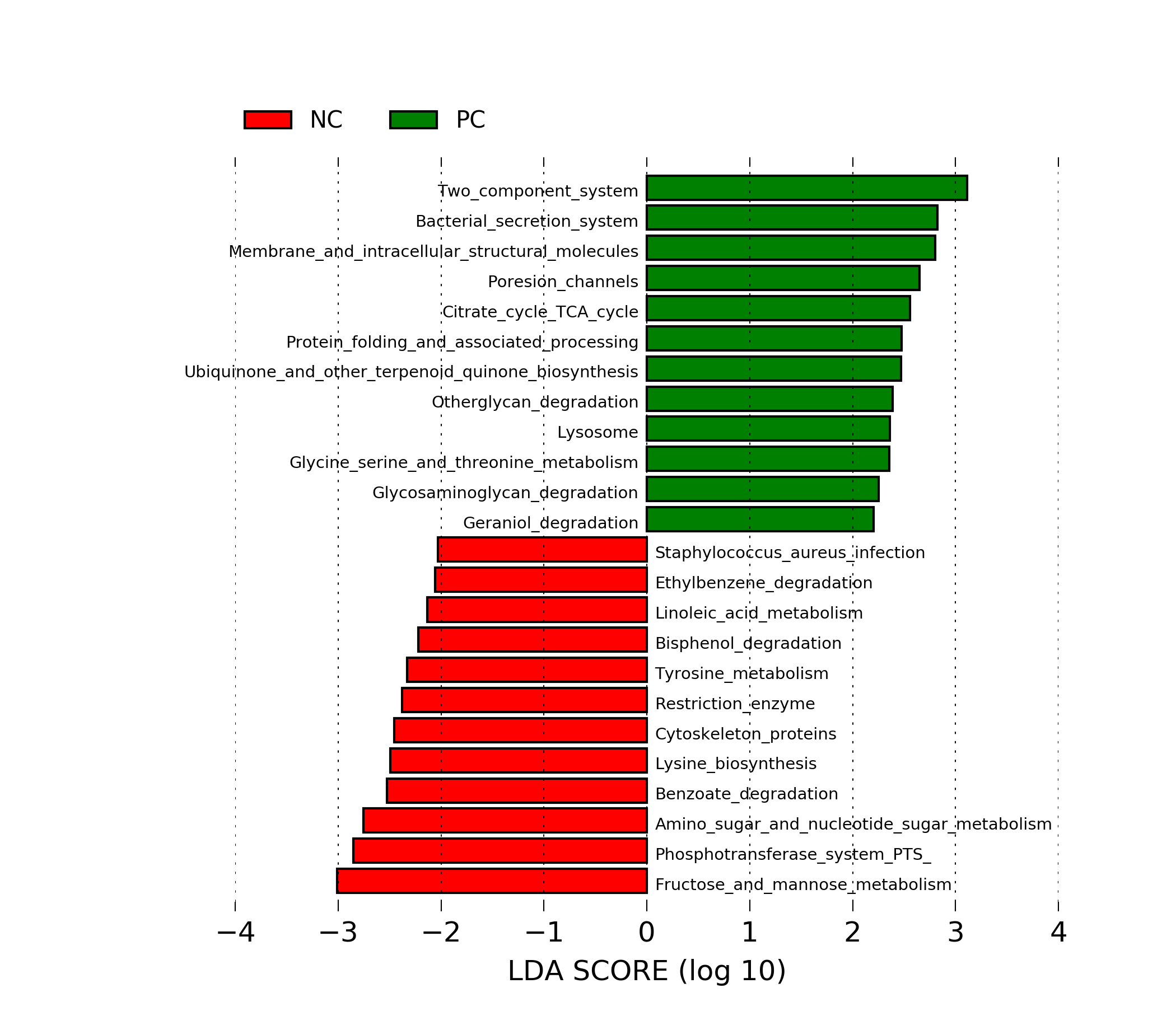


B. PC vs. SF-


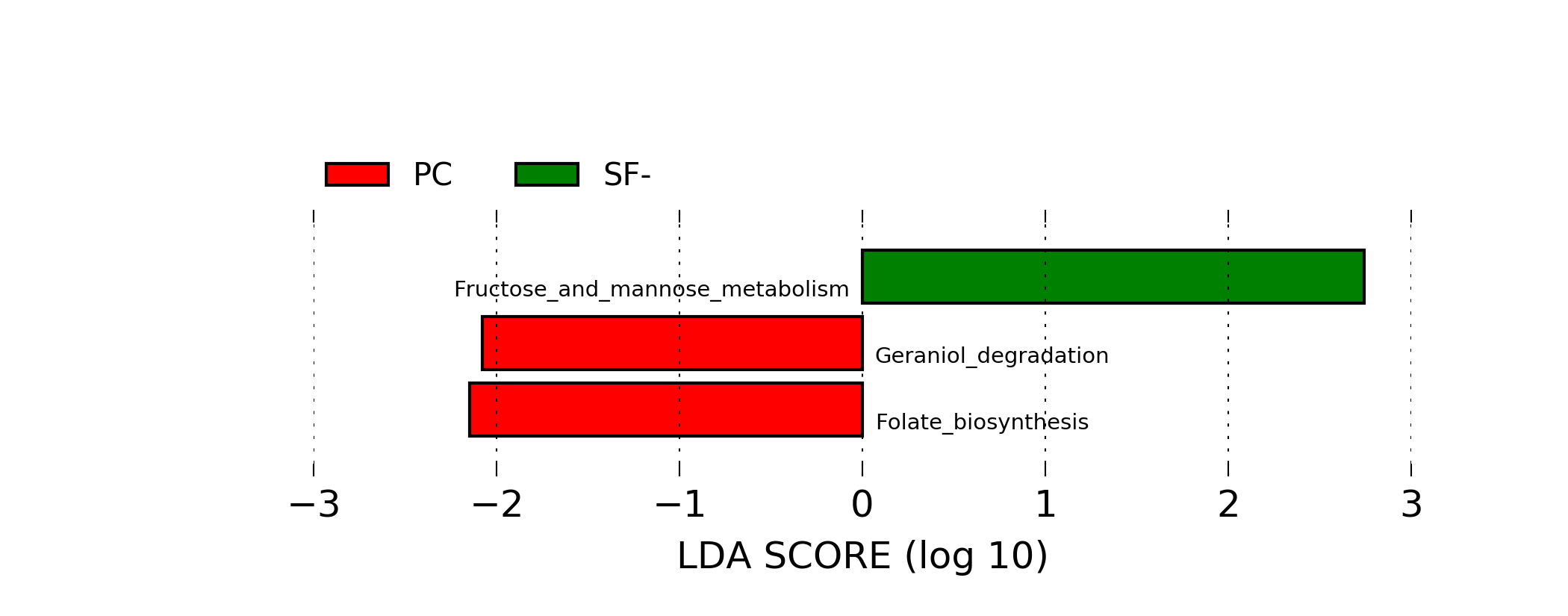


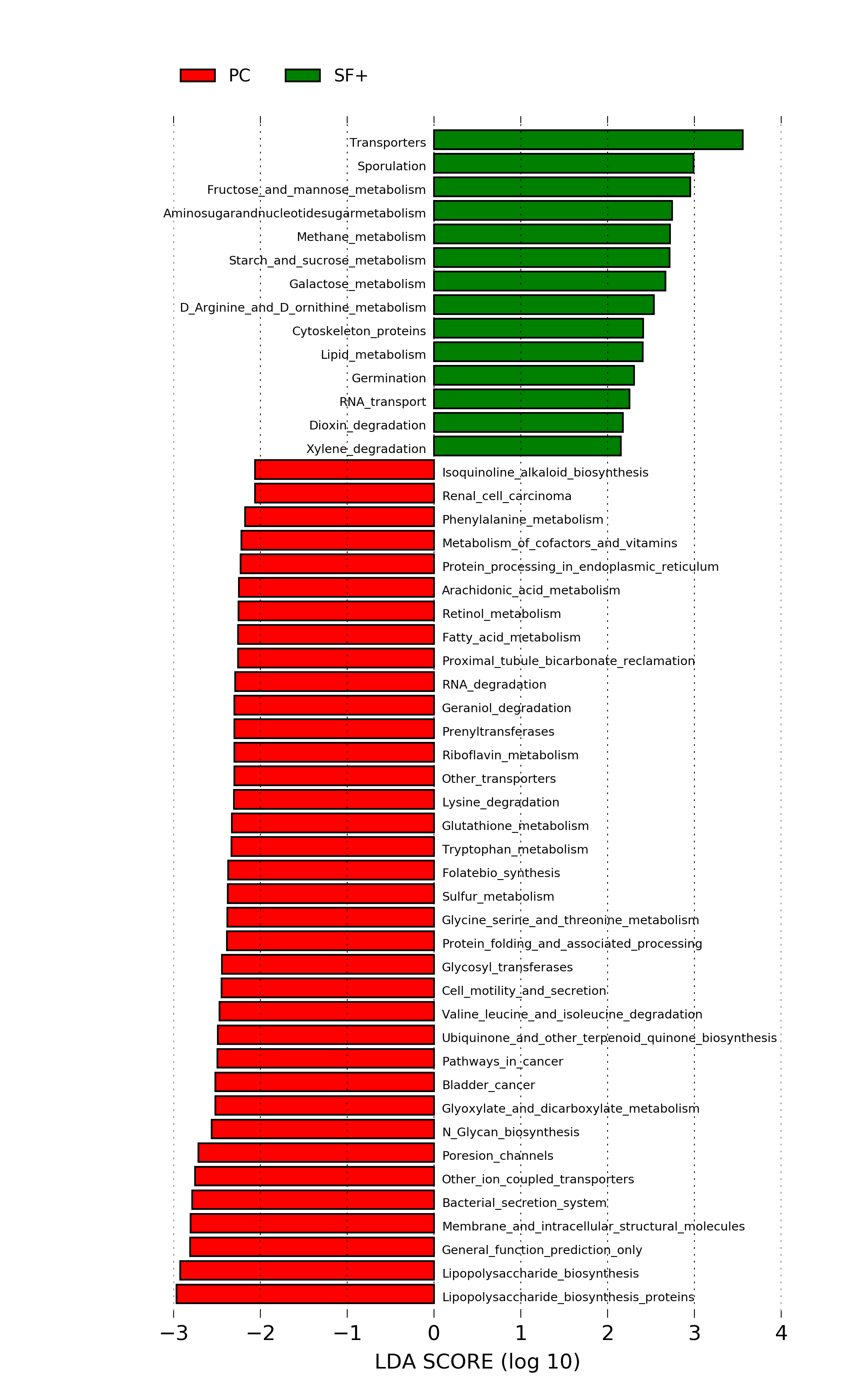


C. PC vs. SF+


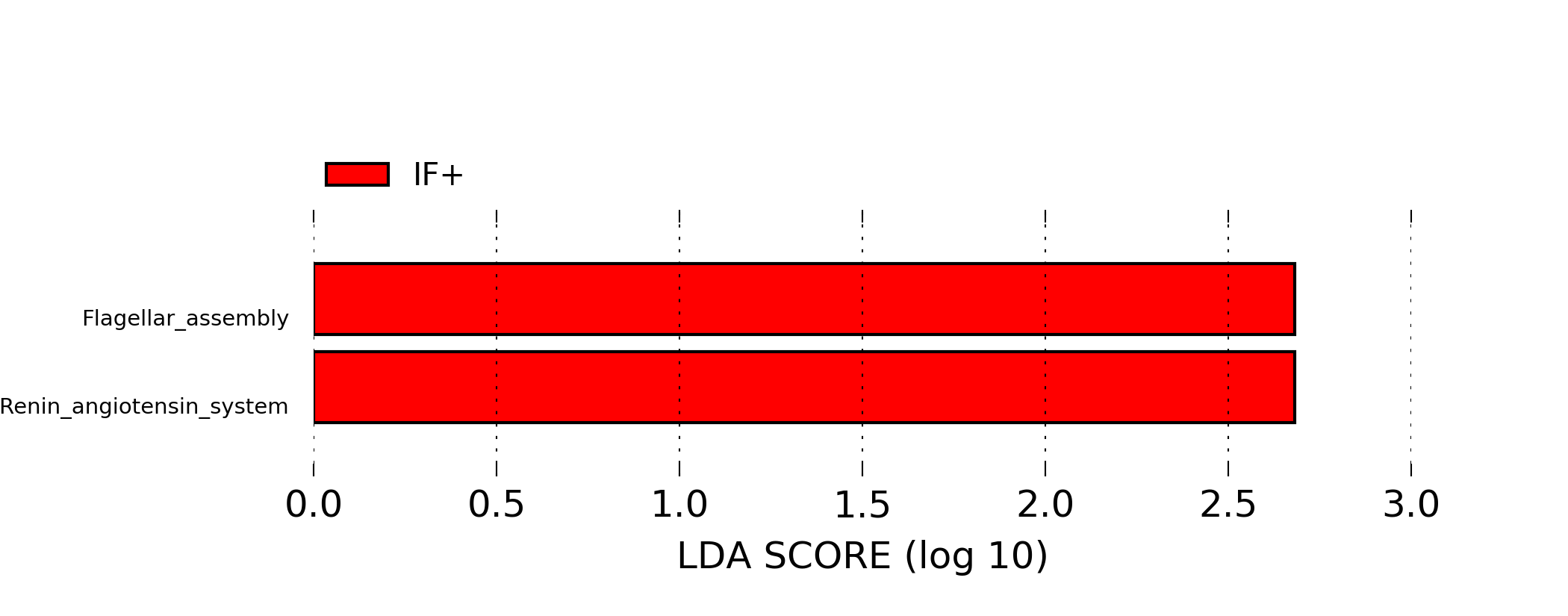

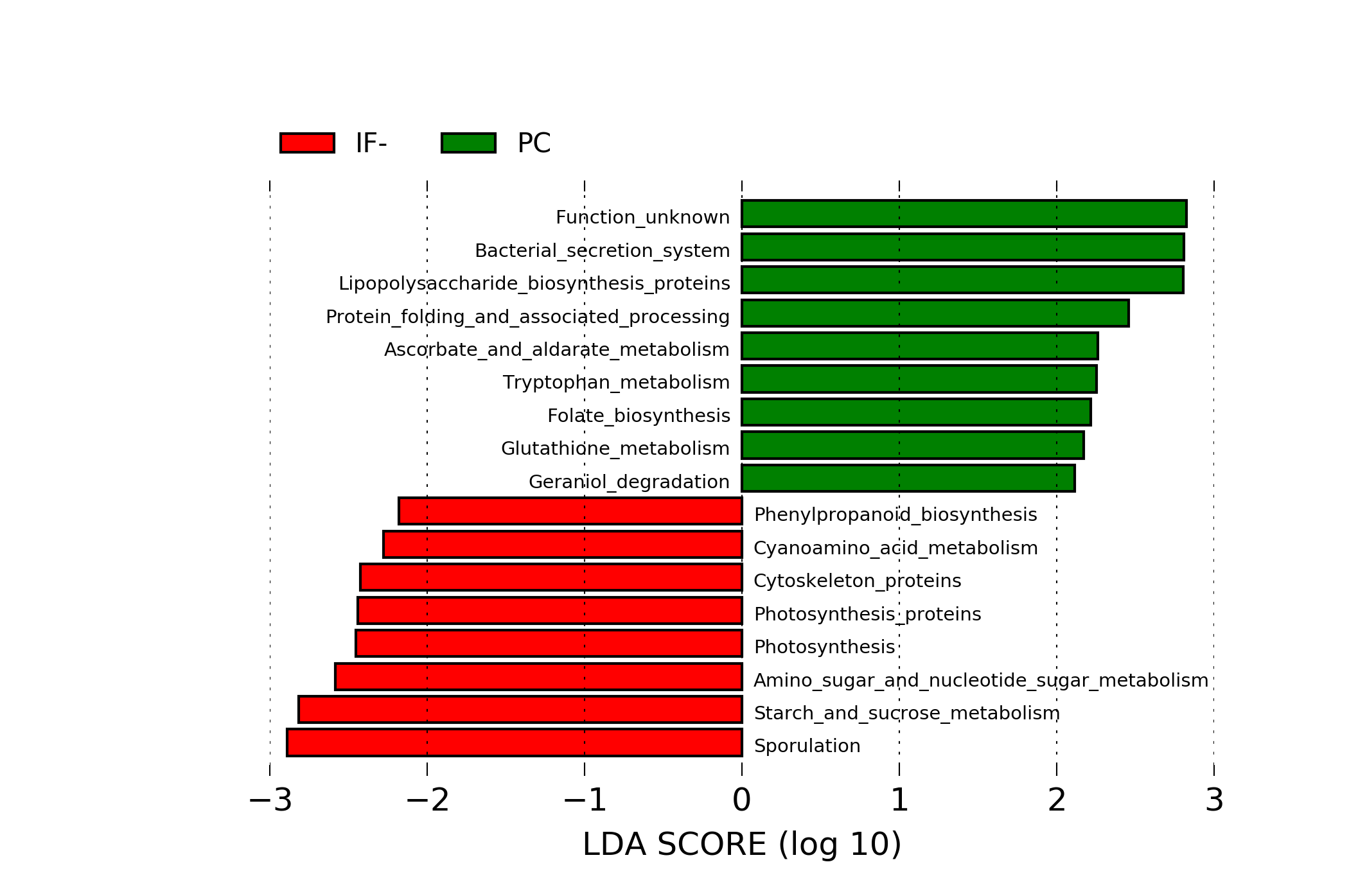


E. PC vs. IF+

D. PC vs. IF-
